# MNM and SNM maintain but do not establish achiasmate homolog conjunction during *Drosophila* male meiosis

**DOI:** 10.1371/journal.pgen.1008162

**Published:** 2019-05-28

**Authors:** Michael Shoujie Sun, Joe Weber, Ariane C. Blattner, Soumya Chaurasia, Christian F. Lehner

**Affiliations:** Institute of Molecular Life Science (IMLS), University of Zurich, Zurich, Switzerland; Stowers Institute for Medical Research, UNITED STATES

## Abstract

The first meiotic division reduces genome ploidy. This requires pairing of homologous chromosomes into bivalents that can be bi-oriented within the spindle during prometaphase I. Thereafter, pairing is abolished during late metaphase I, and univalents are segregated apart onto opposite spindle poles during anaphase I. In contrast to canonical meiosis, homologous chromosome pairing does not include the formation of a synaptonemal complex and of cross-overs in spermatocytes of *Drosophila melanogaster*. The alternative pairing mode in these cells depends on *mnm* and *snm*. These genes are required exclusively in spermatocytes specifically for successful conjunction of chromosomes into bivalents. Available evidence suggests that MNM and SNM might be part of a physical linkage that directly conjoins chromosomes. Here this notion was analyzed further. Temporal variation in delivery of *mnm* and *snm* function was realized by combining various transgenes with null mutant backgrounds. The observed phenotypic consequences provide strong evidence that MNM and SNM contribute directly to chromosome linkage. Premature elimination of these proteins results in precocious bivalent splitting. Delayed provision results in partial conjunction defects that are more pronounced in autosomal bivalents compared to the sex chromosome bivalent. Overall, our findings suggest that MNM and SNM cannot re-establish pairing of chromosomes into bivalents if provided after a chromosome-specific time point of no return. When delivered before this time point, they fortify preformed linkages in order to preclude premature bivalent splitting by the disruptive forces that drive chromosome territory formation during spermatocyte maturation and chromosome condensation during entry into meiosis I.

## Author summary

Meiosis is a special cell division that occurs in two steps, meiosis I and II. It converts diploid into haploid cells which can be used as gametes for sexual reproduction where two gametes from opposite sexes, a sperm and an oocyte, fuse to generate the zygote, the first diploid cell of the next generation. Before the first meiotic division, the two parental copies of each chromosome need to be paired. The resulting bivalents are then integrated in a bipolar fashion into the spindle which separates the chromosome pairs apart and distributes the copies regularly onto opposite spindle poles during the first meiotic division. In males of the fly *Drosophila melanogaster*, the pairing of homologous chromosomes into bivalents is strikingly distinct from the canonical mode, as it does not include formation of a synaptonemal complex and of cross-overs. The proteins MNM and SNM are known to be required specifically for the alternative mode of homolog conjunction in *D*. *melanogaster* spermatocytes. Here we address important questions concerning MNM/SNM function. Are these proteins indeed components of a chromosome glue? If so, how is this glue applied so that exclusively appropriate partner chromosomes are linked? By analyzing the consequences of experimental changes in the program of *mnm* and *snm* expression during spermatocyte maturation, we arrive at a proposal suggesting that MNM and SNM might be part of an indiscriminate glue which is applied in a temporally controlled manner to prevent inappropriate chromosome linkages on the one hand and premature bivalent separation on the other hand.

## Introduction

In preparation for the first meiotic division, homologous chromosomes are paired up into bivalent chromosomes. Pairing into bivalents is required for their bi-polar orientation within the spindle during prometaphase of meiosis I (MI). However, once all bivalents have reached bi-orientation, the ties between homologous chromosomes need to be severed during late metaphase I for reductional homolog segregation onto opposite spindle poles in anaphase I.

How the initial contacts between homologs are established before MI remains poorly understood. During canonical meiosis, pairing culminates with formation of a synaptonemal complex (SC), a conspicuous regular structure that crosslinks homologous chromosomes. As the SC is only a transient structure, additional linkage is required for maintenance of homologs in pairs until onset of anaphase I. These back-up ties result from cross-overs (COs), which are generated by developmentally programmed recombination between homologous sister chromatids, in combination with sister chromatid cohesion within the chromosome arm regions distal from the CO sites. Interestingly, beyond this canonical pairing mode, strikingly different alternatives have evolved. In *Drosophila melanogaster* and other higher dipteran flies, for example, homolog pairing before MI does not include formation of SCs and COs in males [1, 2], while pairing is canonical during female meiosis in these species [3]. Achiasmate meiosis appears to have evolved independently at least 25 times, and different types have been described in diverse evolutionary lineages [4, 5].

Homolog pairing during the achiasmate meiosis in *D*. *melanogaster* spermatocytes is known to involve an alternative homolog conjunction (AHC) system. Genetic approaches have led to the identification of three genes, *modifier of mdg4 in meiosis (mnm*), *stromalin in meiosis*/*SA-2 (snm)* and *teflon (tef)*, that are specifically required for AHC in spermatocytes [6–8]. Loss of function mutations in these genes result in chromosome missegregation during MI exclusively in males. In *mnm* and *snm* mutant males, both sex chromosomes and autosomes are distributed randomly during MI [6]. In contrast, only autosomes are missegregated in *tef* mutant males [7].

The *tef* gene is predicted to encode a protein with three C2H2-type zinc fingers [8]. While transgenes coding for a TEF-EGFP fusion proteins were shown to restore normal MI segregation in *tef* mutants, it has not been possible to detect the TEF-EGFP product expressed from these transgenes [8]. Therefore, the dynamics of TEF expression and its intracellular localization during spermatogenesis have yet to be clarified.

SNM is a distant relative of the stromalins (SCC3/SA/STAG protein family) [6]. Stromalins usually function as subunits of cohesin complexes which make crucial contributions to chromosome organization during interphase and M phases [9]. However, absence of co-localization with core cohesin components has indicated that SNM does not function as a cohesin subunit [6]. MNM is translated from a specific transcript of the highly complex *mod(mdg4)* locus [6, 10]. MNM has an N-terminal BTB/POZ domain that is shared among almost all of the 31 distinct protein products predicted to be expressed from the various *mod(mdg4)* transcripts [10, 11]. In addition, MNM has a unique C-terminal Zn-finger domain of the FLYWCH type. Both domains within MNM are predicted to mediate protein-protein interactions [12]. MNM and SNM accumulate in early spermatocytes where they are strongly enriched in multiple subnucleolar foci [6]. At the start of MI, these foci coalesce into a single prominent spot on the sex chromosome bivalent [6]. Although the X and Y chromosomes are strongly heteromorphic in *D*. *melanogaster*, they both harbor rDNA gene clusters which function as sex chromosome pairing centers during male MI [13, 14]. Immunolabeling combined with fluorescent in situ hybridization (FISH) has clearly demonstrated that the prominent MNM/SNM spot observed on the XY bivalent during MI is closely associated with the rDNA loci of the sex chromosomes [6, 14]. Apart from the strong dot on the XY bivalent, far weaker MNM/SNM signals were observed on autosomal bivalents [6] which rely on euchromatic homology for pairing [2, 15]. Interestingly, the association of MNM and SNM with all bivalents is rapidly lost at the onset of anaphase I in a separase-dependent manner [6, 16]. While present evidence is consistent with the notion that MNM and SNM function as proteinaceous glue that conjoins chromosomes into bivalents, this possibility is far from proven and many crucial questions remain to be answered.

Some of the open questions are accentuated by the dynamics of chromosome pairing during *D*. *melanogaster* spermatogenesis [2]. Spermatogenesis starts with an asymmetric division of a germ line stem cell. The differentiating daughter cell progresses through four transit-amplifying cell cycles with incomplete cytokinesis, resulting in a cyst of 16 interconnected spermatocytes. Spermatocytes grow and mature during progression through the stages S1—S6 [17] before entering into the meiotic divisions. Chromosome pairing into bivalents is completed rapidly in early spermatocytes, as revealed by analyses with a lacO/lacI-GFP system [18]. With this system, a single lacI-GFP dot indicates homolog pairing in cells homozygous for an autosomal lacO array. While only around 50% of the cells displayed homolog pairing during the gonial amplification cycles before S1, about 95% of the cells had paired homologous lacO arrays during S1/S2a [18]. Strikingly, a few hours later around the S2b/S3 transition, pairing was no longer observed for any of 14 distinct euchromatic lacO array loci analyzed [18]. Moreover, beyond the loss of homolog pairing, even sister chromatid cohesion was no longer detectable except in centromeric regions [18, 19]. A similar dynamic of transient homolog pairing was also observed with most FISH probes targeting heterochromatic satellites, including several within pericentromeric regions [19]. The eventual separation of homologous satellite regions was also accompanied by sister chromatid separation in several cases [19].

The dramatic loss of homolog pairing and sister cohesion that starts during stage S2b is accompanied by the process of chromosome territory formation, where the XY bivalent as well as the bivalents formed by the two large autosomes, chromosome 2 (chr2) and chromosome 3 (chr3), are separated apart from each other within the spermatocyte interphase nucleus in a condensin II-dependent manner [2, 20]. The main purpose of this spatial chromatin re-organization into chromosome territories is presumably the breaking up of associations between non-homologous chromosomes. Non-homologous associations that persist until prometaphase I compromise can give rise to missegregation.

Non-homologous associations are prominent in *D*. *melanogaster* cells. The large centromere-proximal heterochromatin regions of all chromosomes, which are brought into close proximity during anaphase in mitotically proliferating cells, usually remain tightly associated within a common chromocenter throughout interphase. During progression through mitotic division cycles, the chromocenter is dissociated when chromosomes condense at the start of M phase. Beyond chromocenter dissociation, chromosome condensation at the start of mitotic divisions also resolves homolog pairing. Somatic pairing of homologous euchromatic chromosome arm regions during interphase is actually extensive in *D*. *melanogaster* cells [21–23]. For the success of meiosis, spermatocytes have to resolve non-homologous associations in a way that does not also disrupt all homologous associations in parallel. Accordingly, the AHC proteins might act to limit collateral damage on homolog pairing and sister chromatid cohesion, which evidently accompanies the disruption of non-homologous associations during chromosome territory formation. In addition, these proteins might also be required at the start of the first meiotic division for inhibition of premature bivalent splitting by chromosome condensation and spindle forces. In *mnm* and *snm* mutants, initial homolog pairing as well as territory formation does not appear to be affected [6]. However, an abnormal expansion of chromosome territories was noted well before the onset of the first meiotic division during which premature bivalent separation into univalents is plainly apparent in these mutants [6, 24].

Here, for an analysis of the temporal phases during which MNM and SNM need to be present in spermatocytes for successful meiosis, we have altered their expression program. By introducing appropriate transgenes into *mnm* and *snm* mutant backgrounds, we have generated genotypes expressing these AHC proteins during either only the early or only the late stages of spermatocyte maturation, or also continuously as in wild-type. We report that early provision of MNM or SNM until chromosome territories have formed is not sufficient. Univalents instead of bivalents are present at MI onset when MNM and SNM are not present during the late stages. Interestingly, late provision of MNM and SNM after chromosome territories have formed is partially sufficient with a pronounced chromosome-specific bias. Compared to the large autosomal bivalents, the sex chromosome bivalent is less dependent on an early presence of MNM and SNM.

## Results

### Rescue of *mnm* and *snm* mutants by transgenic MNM-EGFP and SNM-EGFP expression

**Fig 1:**
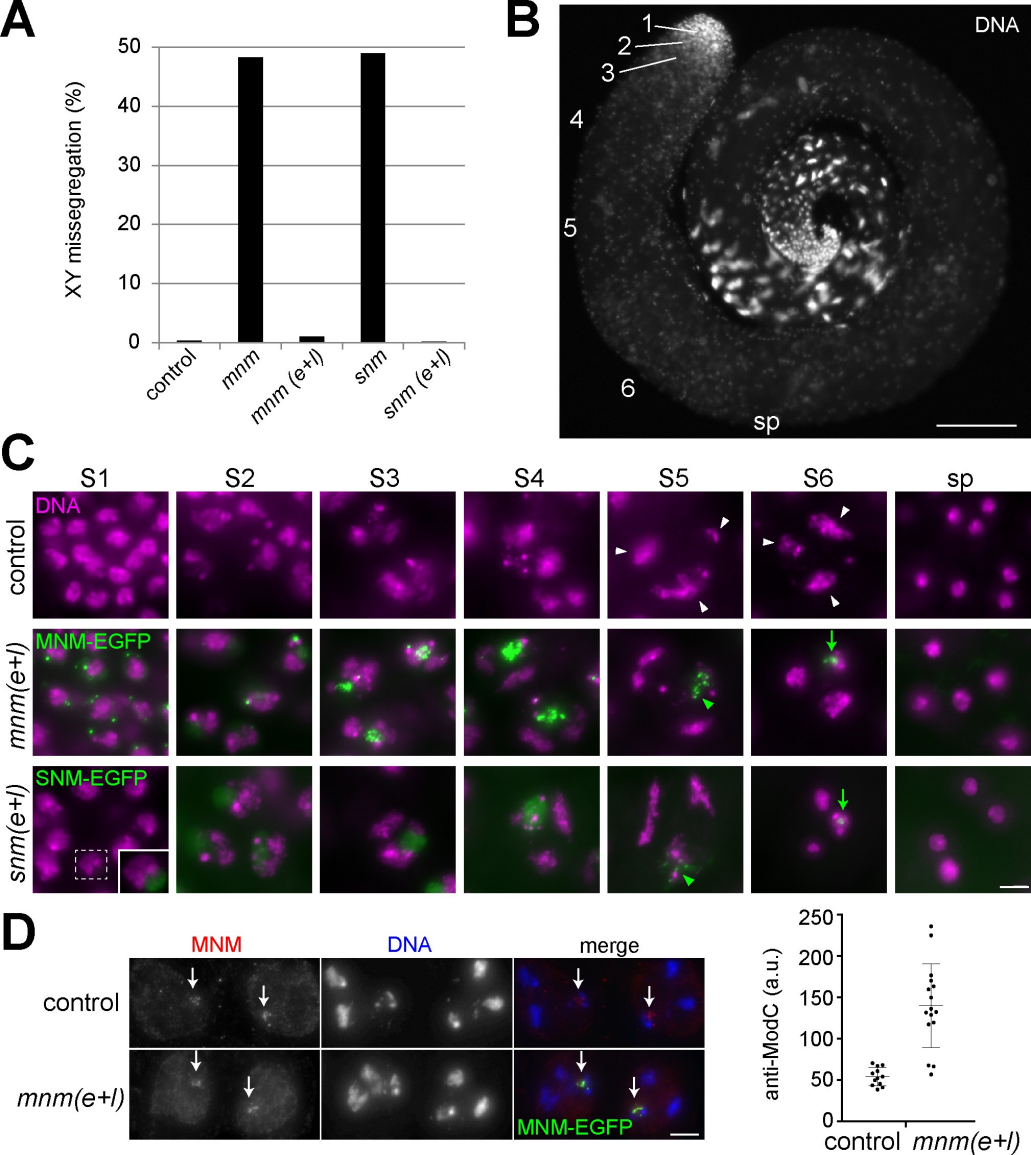
Function and expression of MNM-EGFP and SNM-EGFP in testis. **(A)** Missegregation of sex chromosomes during male meiosis was analyzed in various genotypes by scoring sex and presence of a marked Y chromosome (*B*^*s*^*Yy*^*+*^) in adult F1 progeny. The analyzed genotypes were *w*^*1*^ (control), *mnm*^Z3-3298^/*mnm*^Z3-5578^ (*mnm*), *mnm* with *bamP-GAL4-VP16* and *UASt-mnm-EGFP* (*mnm(e+l)*), *snm*^Z3-0317^/*snm*^Z3-2138^ (*snm*), and *snm* with *bamP-GAL4-VP16* and *UASt-snm-EGFP* (*snm(e+l)*). Expression of *mnm-EGFP* and *snm-EGFP* in *mnm* and *snm*, respectively, corrected the random sex chromosome segregation observed in these null mutants back to regular segregation comparable to controls. **(B)** Whole mount preparation labeled with a DNA stain for illustration of the spatial arrangement of the spermatocyte maturation stages within the coiled epithelial testis tube. The approximate positions of spermatocytes in the stages S1 to S6 are indicated by corresponding numbers, as well as that of early postmeiotic spermatids (sp). Note that the correlation between spatial position and developmental stage is not without exceptions in particular for the advanced stages. **(C)** Regions from testis squash preparations with cells at the indicated stages obtained from the different genotypes. White arrowheads indicate the three major chromosome territories that are most clearly apparent in the late S5 and S6 spermatocyte nucleus after their initial formation already at S2b stage. Green arrowheads indicate the subnucleolar foci formed by both MNM-EGFP and SNM-EGFP most prominently in S5 spermatocytes. During the S6 stage, in parallel with chromosome condensation in preparation for the first meiotic division, these foci condense into a single EGFP dot associated with the sex chromosome bivalent (green arrow). Subnucleolar foci are not yet apparent at the start of MNM-EGFP and SNM-EGFP accumulation. To reveal the weak initial, diffuse nucleolar localization of SNM-EGFP, an S1 spermatocyte nucleus (dashed rectangle) is shown in the inset after enhancement of SNM-EGFP signals. **(D)** The expression level of endogenous MNM in control was compared with that of MNM-EGFP in *mnm(e+l)* by staining testis squash preparations with anti-MNM followed by quantification of signal intensities in the dot associated with the XY bivalent (arrows) in prometaphase cells (n = 12 and 16, respectively). Dot plot (with average and s.d.) indicates on average 2.6 fold overexpression in *mnm(e+l)* (p < 0.0001, t test). Bars = 100 μm (B), 4 μm (C) and 8 μm (D).

To manipulate the temporal program of *mnm* and *snm* expression during spermatocyte maturation we made use of the GAL4/UAS system. We generated lines with *UASt* transgenes allowing expression of MNM or SNM with and without EGFP extensions (*UASt-mnm*, *UASt-EGFP-mnm*, *UASt-mnm-EGFP*, *UASt-snm*, *UASt-EGFP-snm*, and *UASt-snm-EGFP*). To assess functionality we expressed the *UASt* transgenes in flies trans-heterozygous for mutations in *mnm* or *snm*, respectively. The selected mutant *mnm* and *snm* alleles are null alleles based on genetic tests [6]. *bamP-GAL4-VP16* was used to drive germline-specific *UASt* transgene expression. Moreover, a dominantly marked Y chromosome was crossed into the males, permitting an analysis of irregularities in sex chromosome transmission onto the next generation. As expected [6], sex chromosome segregation occurred randomly in *mnm* and *snm* mutant males. But *bamP-GAL4-VP16*-driven expression of the *UASt* transgenes in these mutants prevented sex chromosome missegregation largely or even completely (S1 Table). Transgenes driving C-terminally EGFP-tagged versions, *UASt-mnm*-EGFP and *UASt-snm-EGFP*, that restored sex chromosome missegregation in the mutants back to wild-type level (Fig 1A) were selected for further experiments.

To characterize the expression pattern resulting from *bamP-GAL4-VP16*-driven expression of *UASt-mnm-EGFP* and *UASt-snm-EGFP*, we analyzed whole mount and squash preparations of testes (Fig 1B and 1C). As expected based on the known pattern of *bamP-GAL4-VP16* expression [25], GFP expression was not observed in somatic cells. In the germline, GFP signals were also not yet detectable in stem cells and gonial cells except weakly during the last gonial division cycle. GFP signal intensities increased strongly during the initial spermatocyte stage (Fig 1C). In early S1 spermatocytes [17], MNM-EGFP was detected mainly in one to a few strong dots that were within the nucleus but apparently not associated with chromatin (Fig 1C). In contrast, the initial accumulation of SNM-EGFP was less apparent and occurred diffusely throughout the nucleolus (Fig 1C). During the later stages MNM-EGFP also shifted into the nucleolus. The initial diffuse nucleolar localization of both MNM-EGFP and SNM-EGFP was increasingly transformed into distinct subnucleolar foci (Fig 1C). At the S6 stage, where chromosome territories start to condense and the nucleolus is disassembled in preparation for the first meiotic divisions, the subnucleolar foci of MNM-EGFP and SNM-EGFP started to coalesce into a strong single dot marking the sex chromosome bivalent until onset of anaphase I (Fig 1C). After the meiotic divisions, EGFP signals were no longer detectable within the nuclei of early round spermatids (Fig 1C).

The observed pattern of *UASt-mnm-EGFP* and *UASt-snm-EGFP* expression driven by *bamP-GAL4-VP16* (Fig 1C) appeared to be similar to the endogenous *mnm* and *snm* expression pattern described previously [6]. For further comparison of endogenous and transgenic expression, we performed immunofluorescent labeling with antibodies against MNM. In control testes, these antibodies reveal the expression from the endogenous locus (Fig 1D). In *mnm* mutant testes, these antibodies no longer generate specific signals [6]. Therefore, these antibodies presumably detect exclusively transgene-derived protein in *mnm* mutant testis with *bamP-GAL4-VP16* and *UASt-mnm*-EGFP (*bamP>mnm-EGFP*) (Fig 1D). Quantitative comparison of signal intensities during prometaphase I, a short and unequivocally identifiable stage with a compact dot-like signal on the sex chromosome bivalent, suggested that the level of MNM-EGFP observed in *mnm* mutants with *bamP>mnm-EGFP* was about threefold higher than the level of MNM in wild-type (Fig 1D). In case of SNM, we were unable to obtain immunofluorescent signals that were sufficiently above background for a robust quantification. In the following, the *mnm* null mutants rescued by *bamP>mnm-EGFP* will be designated as *mnm(e+l)* since MNM-EGFP is present from the early until the late spermatocyte stages. Analogously, *snm(e+l)* will be used for *snm* null mutants rescued by *bamP>snm-EGFP*.

Apart from the strong dots on sex chromosome bivalents, far fainter and smaller EGFP specs were detectable on autosome bivalents in *mnm(e+l)* and *snm(e+l)* during meiosis I until anaphase onset, as also reported previously after analyses of the endogenous *mnm* and *snm* expression with antibodies or with transgenes expressing EGFP fusions under control of other promoters [6]. The following descriptions will not comment on these far weaker signals.

### Transient presence of MNM-EGFP in early spermatocytes does not restore *mnm* function

To confine *bamP-GAL4-VP16* driven *UASt-mnm-EGFP* expression to the early stages of spermatocyte maturation we combined it with deGradFP [26] for depletion of MNM-EGFP during the late stages. Protein depletion by deGradFP is achieved by expression of an Nslmb-vhhGFP4 fusion protein which results in polyubiquitination and consequential proteasomal degradation of GFP-tagged proteins. To express Nslmb-vhhGFP4 exclusively during late stages of spermatocyte maturation, we generated a transgene under control of the *betaTub85D* cis-regulatory region [27]. This *betaTub85DP-Nslmb-vhhGFP4* transgene was combined with *bamP-GAL4-VP16* and *UASt-mnm-EGFP* in the transheterozygous *mnm* mutant background, resulting in a genotype designated as *mnm(e)* in the following.

**Fig 2:**
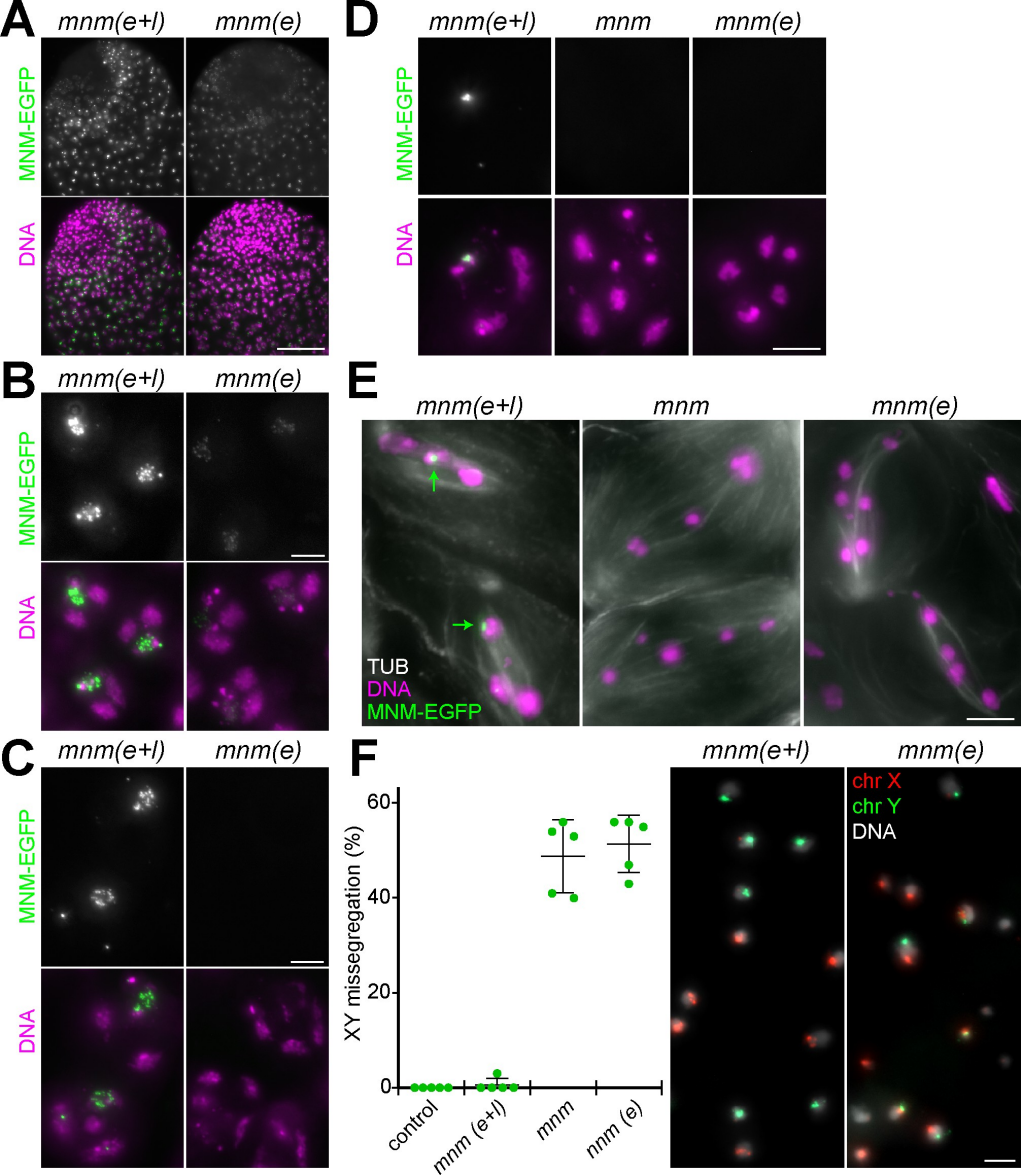
Transient presence of MNM-EGFP in early spermatocytes does not restore *mnm* function. While MNM-EGFP is present in *mnm(e+l)* throughout spermatocyte maturation until late metaphase I, deGradFP was used in *mnm(e)* for MNM-EGFP depletion during the late spermatocytes stages. Expression pattern and phenotypic consequences were analyzed in testis preparations. **(A)** Apical testis regions reveal comparable onset of MNM-EGFP accumulation in late gonial and early spermatocyte cysts in both *mnm(e+l)* and *mnm(e)* although levels are lower in the latter genotype. **(B)** At the S3 stage, MNM-EGFP is still readily detectable and chromosome territories are normal in *mnm(e)*. **(C)** At the S5 stage, MNM-EGFP is no longer detectable and chromosome territories are less compact in *mnm(e)*. **(D)** At the S6 stage, the number of condensing chromosome territories is clearly increased in *mnm(e)*, similar as in *mnm* null mutants. **(E)** During prometaphase I (identified by anti-tubulin labeling), more and smaller chromosome masses are present in *mnm(e)* and *mnm* than in *mnm(e+l)* which displays the characteristic number of three large bivalents (chrXY with MNM-EGFP dot, chr2 and chr3) and one small bivalent (chr4). **(F)** FISH with a red fluorescent probe for chrX and a green fluorescent probe for chrY was used to analyze the rate of sex chromosome missegregation in the indicated genotypes. Normal segregation results in either a red or a green signal per spermatid nucleus, as illustrated with a region from an *mnm(e+l)* cyst. Missegregation results in spermatid nuclei without or with both red and green signals and variable sizes, as illustrated with a region from an *mnm(e)* cyst. n = 5 early spermatid cysts for each genotype. Average and s.d. are indicated. Bars = 50 μm (A) and 5 μm (B-F).

We analyzed testis preparations to assess whether MNM-EGFP is indeed present exclusively during the early spermatocyte stages in *mnm(e)* males. As expected, EGFP signals were clearly present in early spermatocytes (Fig 2A and 2B). However, compared to *mnm(e+l)* these signals were weaker (Fig 2A and 2B), presumably reflecting low level expression of *betaTub85DP-Nslmb-vhhGFP4* already in early spermatocytes. EGFP signal quantification indicated that MNM-EGFP is around 3–4 fold lower in *mnm(e)* compared to *mnm(e+l)*. Considering the estimated level of overexpression in *mnm(e+l)* (Fig 1C), the levels of MNM-EGFP present in early *mnm(e)* spermatocytes should be comparable to the MNM levels in wild-type. At the S3 stage, where chromosome territories are already clearly recognizable, MNM-EGFP was readily detectable also in *mnm(e)* spermatocytes (Fig 2B). Importantly, MNM-EGFP signals dropped sharply thereafter in *mnm(e)* testis. During the S5 stage (Fig 2C), as well as the subsequent stages S6 (Fig 2D) and prometaphase I (Fig 2E), MNM-EGFP was no longer detectable in *mnm(e)*, while in *mnm(e+l)* it persisted within strong subnucleolar foci and later in the characteristic strong dot on the sex chromosome bivalent during prometaphase I.

DNA labeling revealed that chromosome territories became abnormal in *mnm(e)* spermatocytes in parallel with the disappearance of MNM-EGFP. In S5 spermatocytes, territories in *mnm(e)* were not as confined as in *mnm(e+l)* (Fig 2C). The increasing condensation of chromosomes during the S6 stage (Fig 2D) and in prometaphase I (Fig 2E) exposed the territory abnormalities in *mnm(e)* spermatocytes further. Prometaphase I cells were identified after double labeling of spindles with anti-tubulin. During normal prometaphase I, where chromosomes are maximally condensed, three large bivalents (those of chrXY, chr2 and chr3) and a small bivalent (chr4) can ususally be distinguished. In *mnm(e+l)*, we observed the characteristic normal number and pattern of bivalents (Fig 2E). In contrast, an increased number of smaller DNA blobs were observed in *mnm(e)* (Fig 2E). This phenotype was indistinguishable from that observed in *mnm* null mutants (Fig 2D and 2E) [6].

The obvious defect in homolog conjunction observed in *mnm(e)* during prometaphase I is predicted to cause chromosome missegregation during MI. To assess the extent of chromosome missegregation, we performed FISH with a red fluorescent chrX probe and a green fluorescent chrY probe. After normal segregation of chrX and chrY during MI, spermatid nuclei are expected to have either a red or a green signal. In contrast, missegregation of the sex chromosomes will result in nuclei with either both a red and a green signal or no signal. In early spermatid cysts from *mnm(e+l)* males, we observed the expected normal pattern (Fig 2F). In contrast, in *mnm(e)*, nuclei with a pattern of FISH signals indicating missegregation were frequent (Fig 2F). Quantification of the fraction of spermatids with normal FISH signals (either red or green) or abnormal signals (either none or both red and green) revealed minimal sex chromosome missegregation in control and *mnm(e+l)* (Fig 2F), consistent with our initial genetic analyses (Fig 1A). In contrast, sex chromosome segregation was found to be random in *mnm(e)*, as also in *mnm* null mutants (Fig 2F). Random sex chromosome segregation in *mnm* null mutants had already been established earlier by genetic analyses (Fig 1A) [6]. Beyond the pattern of FISH signals, the striking size variation among the nuclei present in spermatid cysts of *mnm(e)* (Fig 2F) and *mnm* null provided further evidence of chromosome missegregation during meiosis.

Overall, the results of our phenotypic analyses with *mnm(e)* males indicate that a provision of MNM exclusively during the early spermatocyte stages is not sufficient for normal chromosome conjunction and segregation during MI. Apparently MNM needs to be present after chromosome territories have formed until the late spermatocyte stages for normal chromosome segregation during MI.

### Transient presence of SNM-EGFP in early spermatocytes does not restore *snm* function

**Fig 3:**
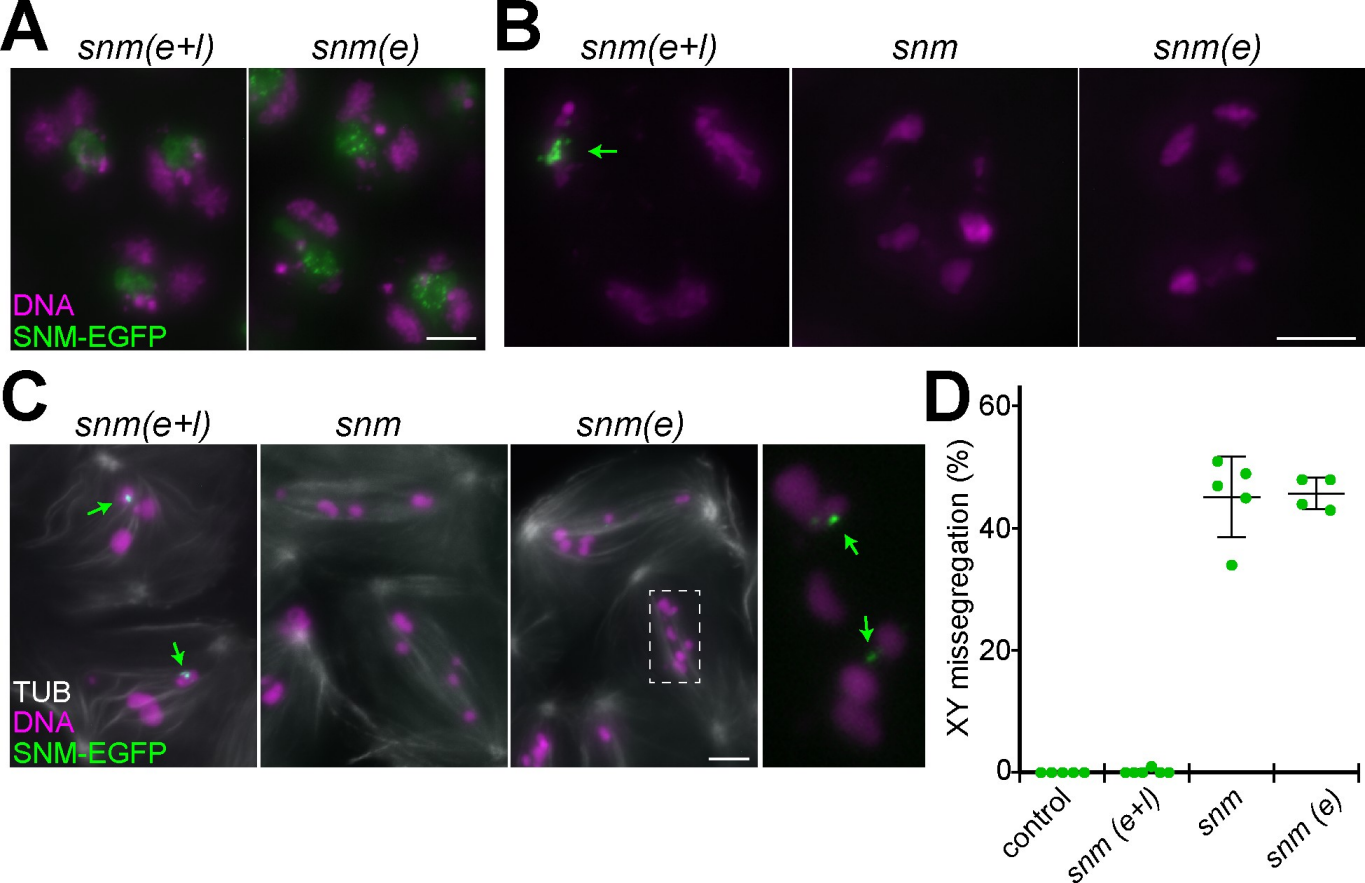
Transient presence of SNM-EGFP in early spermatocytes does not restore *snm* function. While SNM-EGFP is present throughout spermatocyte maturation until late metaphase I in *snm(e+l)*, deGradFP was used for SNM-EGFP depletion during the late spermatocyte stages in *snm(e)*. Expression pattern and phenotypic consequences were analyzed in testis preparations. **(A)** At the S3 stage, SNM-EGFP is detectable at comparable levels in *snm(e+l)* and *snm(e)*, and normal chromosome territories are present. **(B)** At the S6 stage and **(C)** during prometaphase I, SNM-EGFP is barely detectable in *snm(e)*, where more and smaller chromosomes are present compared to *snm* null mutants. In (C), the boxed region is shown at higher magnification with enhanced green signals, revealing residual SNM-EGFP in two dots (arrows) associated with two non-conjoined chromosomes, presumably chrX and chrY. **(D)** The rate of sex chromosome missegregation was assessed in early spermatid cysts of the indicated genotypes after XY FISH. n = 5 cysts for each genotype. Average and s.d. are indicated. Bars = 5 μm (A-C).

The deGradFP method was also applied for the elimination of SNM-EGFP during the late spermatocyte stages. The genotype resulting from combining *betaTub85DP-Nslmb-vhhGFP4* with *bamP-GAL4-VP16* and *UASt-snm-EGFP* in the transheterozygous *snm* mutant background will be designated as *snm(e)* in the following. Microscopic analyses were used to confirm SNM-EGFP elimination in *snm(e)* (Fig 3). In early *snm(e)* spermatocytes, SNM-EGFP signals were present and comparable to those observed in *snm(e+l)* (Fig 3A). However, in late *snm(e)* spermatocytes SNM-EGFP signals were far lower compared to *snm(e+l)* (Fig 3B and 3C). Therefore, SNM-EGFP elimination by deGradFP was clearly successful, but less efficient than that of MNM-EGFP, as indicated by the following observations. In case of *mnm(e)*, MNM-EGFP signals were noticeably decreased already at the onset of expression and absent in prometaphase I (Fig 2). In contrast, in *snm(e)*, SNM-EGFP signals were not obviously reduced already in early spermatocytes and often still detectable during prometaphase I although only very weakly (Fig 3C). Variable deGradFP efficiency with different GFP target proteins, as in case of MNM-EGFP and SNM-EGFP, has been observed before [26]. However, despite some residual SNM-EGFP in *snm(e)*, chromosome conjunction and segregation during MI were clearly defective. As revealed by DNA staining at the late S6 stage (Fig 3B) and during prometaphase I (Fig 3C), chromosomes were more numerous and smaller in *snm(e)* compared to *snm(e+l)* where the normal number of three large and one small bivalent was present. The residual low SNM-EGFP signals that were still present during prometaphase I in *snm(e)* were maximal in two dots associated with two distinct chromosomes (Fig 3C), i.e., most likely the unconjoined chrX and chrY. In contrast, a single strong SNM-EGFP dot was present on the XY bivalent during prometaphase I in *snm(e+l)* (Fig 3C), as observed with anti-SNM in wild-type testis [6]. The chromosome abnormalities observed in *snm(e)* at the onset of MI were comparable to those observed in *snm* null mutants (Fig 3B and 3C). Quantitative analysis of meiotic sex chromosome segregation by FISH (Fig 3D) revealed random segregation in *snm(e)* and *snm* null, while marginal missegregation was detected in *snm(e+l)* and control. We conclude that as in case of MNM, provision of SNM exclusively during the early spermatocyte stages is not sufficient for normal chromosome conjunction and segregation during MI.

### Delayed MNM-EGFP expression rescues *mnm* mutants partially

**Fig 4:**
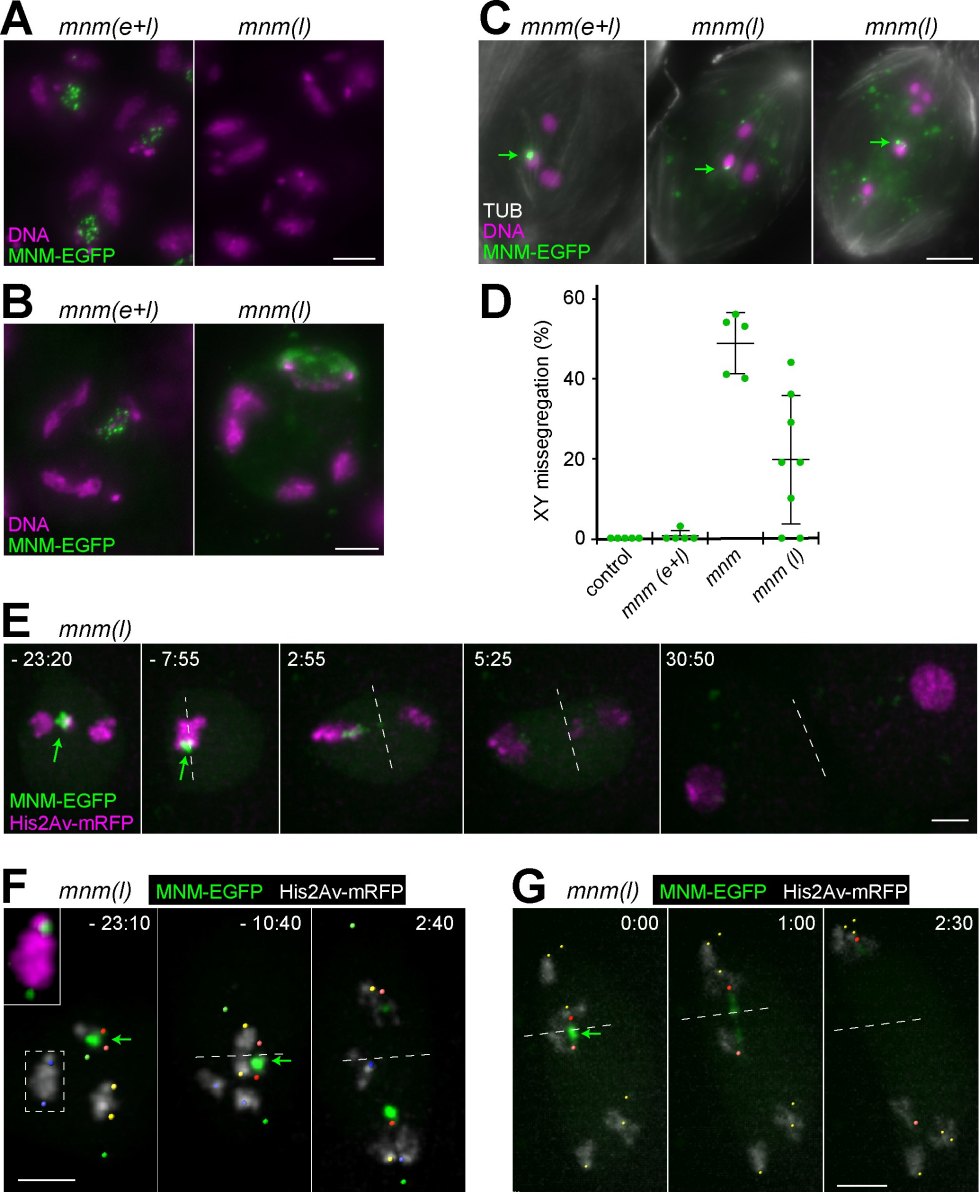
Delayed MNM-EGFP expression rescues *mnm* mutants partially. To delay expression of MNM-EGFP until the late spermatocytes stages, a *betaTub85DP-mnm-EGFP* transgene was introduced into *mnm* null mutants. Expression pattern and phenotypic consequences were analyzed in testes isolated from the resulting *mnm(l)* genotype. **(A)** At the S3 stage, MNM-EGFP is not yet detectable in *mnm(l)* in contrast to *mnm(e+l)*. **(B)** At the S6 stage, however, MNM-EGFP is present in both *mnm(l)* and *mnm(e+l)*. **(C)** During prometaphase I, MNM-EGFP levels are higher in *mnm(l)* compared to *mnm(e+l)*. Throughout the *mnm(l)* cell, additional weaker dots without chromosome associations are distributed apart from a most prominent MNM-EGFP dot on the chrXY bivalent (arrows). Chromosome appearance is variable in *mnm(l)*. As in *mnm(e+l)*, the normal pattern with three major and one small bivalent is present in some *mnm(l)* prometaphase I cells (middle panel). In others (right panel), chromosome masses are more numerous and smaller. **(D)** The rate of sex chromosome missegregation was assessed in early *mnm(l)* spermatid cysts (n = 8) after XY FISH. For comparison, data from control, *mnm(e+l)* and *mnm* (see Fig 2F) is included. Average and s.d. are indicated. **(E-G)** Time lapse imaging was used for an analysis of progression through MI in *mnm(l)* expressing histone His2Av-mRFP and the centromere marker Cenp-A/Cid-EGFP. The latter signals are far weaker than the strong MNM-EGFP dot on the chrXY bivalent; hence at display settings below excessive saturation of this dot, centromere signals are not visible. **(E)** Still frames from an *mnm(l)* spermatocyte with almost normal progression through MI. Equatorial plane indicated by the dashed lines. Time (minutes: seconds) is given relative to the last metaphase I frame (t = 0). Normal bivalents congress into the metaphase plate and are separated apart onto opposite spindle poles during anaphase concomitant with disappearance of the MNM-EGFP dot from the chrXY bivalent which separates with a slight delay. **(F)** An *mnm(l)* spermatocyte in which some bivalents separate prematurely. Centromere positions detected after enhancement of green signals (as in the inset displaying the boxed bivalent) are indicated by colored dots: chrXY (red), large autosome 1 (yellow), large autosome 2 (blue), and chr4 (green). While the chrXY bivalent and one large autosome bivalent (yellow dots) displayed normal behavior, the other large autosomal bivalent (blue dots) separated prematurely during prometaphase I. Moreover, univalents instead of bivalents where present already at the start of MI in case of chr4. **(G)** An *mnm(l)* spermatocyte in which all but the chrXY bivalent have separate prematurely. Bars = 5 μm (A-C) and 3 μm (E-G).

To delay expression of MNM-EGFP until the late stages of spermatocyte maturation, we generated a *betaTub85DP-mnm-EGFP* transgene and crossed it into the transheterozygous *mnm* mutant background. The resulting genotype will be designated as *mnm(l)*. Microscopic analyses were performed to determine the temporal program of MNM-EGFP expression in *mnm(l)* testis. At the S3 stage, where chromosome territories have already formed, MNM-EGFP was not yet detectable in *mnm(l)*, while it was clearly present in *mnm(e+l)* (Fig 4A). Subsequently MNM-EGFP accumulation started in *mnm(l)* in the nucleolus. Eventually some MNM-EGFP dots also appeared outside of the nucleolus, dispersed within the cytoplasm. At the early S6 stage (Fig 4B) such cytoplasmic MNM-EGFP dots were already detectable in *mnm(l)* apart from the stronger nucleolar signals. In contrast, cytoplasmic MNM-EGFP dots were not apparent in *mnm(e+l)* (Fig 4B). Later in *mnm(l)* during prometaphase I (Fig 4C) dispersed MNM-EGFP dots without chromosome association were even more numerous and stronger. However, these dots were usually still weaker than the most intense MNM-EGFP dot which was closely associated with a bivalent, presumably the sex chromosome bivalent (Fig 4C). The intensity of this bright chromosomal MNM-EGFP dot was variable between spermatocytes, reaching levels above that of the dot on the chrXY bivalent in *mnm(e+l)*. We assume that the non-chromosomal MNM-EGFP dots within the cytoplasm of *mnm(l)* arise because the *betaTub85D* regulatory region drives very strong expression during the final spermatocyte stages.

The analysis of chromosome number and size in prometaphase I (Fig 4C) revealed considerable phenotypic variability in *mnm(l)*. While some cysts had spermatocytes displaying the normal number of four masses of DNA staining (three large and one small bivalent), other cysts had cells with clearly too many distinct chromosomal blobs (Fig 4C). In prometaphase I cells with a normal pattern of bivalents, the most prominent MNM-EGFP dot was on a large chromosomal DNA mass (Fig 4C), most likely representing a normally conjoined chrXY bivalent. Interestingly, such an apparently normal chrXY bivalent characterized by the most prominent MNM-EGFP dot was also present in the large majority of prometaphase I cells with an abnormal chromosome pattern, raising the possibility that late provision of MNM-EGFP is more detrimental for autosomal bivalents compared to sex bivalents. Only a minority of prometaphase I cells had two prominent MNM-EGFP dots that were on two distinct DNA blobs, indicating an occasional failure of sex chromosome conjunction.

A quantitative analysis of meiotic sex chromosome segregation by XY FISH clearly confirmed the phenotypic variability between *mnm(l)* cysts (Fig 4D). Among the eight early postmeiotic cysts analyzed, the frequency of chrXY missegregation ranged from 50% (i.e., random segregation as in *mnm* null mutants) to zero (as in wild-type control).

For further characterization of MI chromosome segregation in *mnm(l)*, we applied time lapse imaging using spermatocytes expressing histone H2Av-mRFP (His2Av-mRFP) and Cenp-A/Cid-EGFP for labeling of chromosomes and centromeres, respectively. Analogous analyses in wild-type and *mnm* null mutant spermatocytes have been described recently [24]. In wild-type MI, chromosome condensation around nuclear envelope breakdown (NEBD) converts the bivalents into compact blobs. The His2Av-mRFP marker reveals the large bivalents (chrXY, chr2 and chr3) readily but not that formed by the small dot chromosome (chr4) [24]. Rapid saltatory movements during prometaphase I accompany the bi-polar integration of bivalents into a compact metaphase I plate that remains stable for 15–20 minutes until bivalents split in anaphase I [24]. In contrast, in *mnm* null mutants, bivalents are separated prematurely into univalents. While some bivalents were still intact at NEBD in *mnm* null mutants, these were all very rapidly converted into univalents as soon as spindle forces started to act on kinetochores during prometaphase I [24]. After a temporally extended phase with saltatory movements in *mnm* null mutants, most univalents eventually reached stable positions preferentially near the poles, followed by anaphase onset and exit from MI [24].

Compared to wild type and *mnm* null mutants [24], the *mnm(l)* phenotype was observed to be intermediate and more variable (Fig 4E–4G). The *mnm(l)* cells, which expressed the fluorescent markers His2Av-mRFP and Cid-EGFP as in our previous analyses [24], produced MNM-EGFP in addition. This MNM-EGFP expression compromised centromere detection to some extent. MNM-EGFP generated an increased diffuse nucleoplasmic signal, as well as some dispersed non-chromosomal dots and usually a very strong dot on the chrXY bivalent. In Fig 4E–4G, the centromere signals are therefore not apparent (except for Fig 4F inset) because their visualization requires display settings resulting in excessive saturation of the MNM-EGFP dot on the chrXY bivalent. However, in Fig 4F and 4G, we have marked centromere positions by small colored spheres. Progression through MI was analyzed in a total of 15 *mnm(l)* cells from five different cysts. In eight of the 15 *mnm(l)* cells, an apparently normal MI was observed (Fig 4E, S1 Movie). His2Av-mRFP signals revealed the presence of normal large bivalents congressing into a stable metaphase plate and splitting at the onset of anaphase (Fig 4E, S1 Movie). The intensity of the very strong MNM-EGFP dot on the chrXY bivalent decreased rapidly during anaphase (Fig 4E, S1 Movie). However, segregation of chrX and chrY towards opposite poles started already when considerable amounts of the overexpressed MNM-EGFP were still associated with the sex chromosomes, resulting in stretching of the MNM-EGFP dot and a slight lag of sex chromosome separation (Fig 4E, S1 Movie). In the remaining seven of the analyzed *mnm(l)* cells that progressed through MI, one of the large autosomal bivalents (Fig 4F) or all autosomal bivalents (Fig 4G, S2 Movie) were separated prematurely into univalents. The premature bivalent splitting occurred after NEBD but well ahead of the metaphase to anaphase I transition, presumably as a result of pulling forces ensuing from interactions with spindle microtubules. Interestingly, in all *mnm(l)* cells with premature splitting of autosomal bivalents, the sex chromosome bivalent displayed a normal behavior, i.e., stable biorientation within the equatorial plane during metaphase followed by separation at anaphase onset (Fig 4F and 4G, S2 Movie). Time lapse imaging therefore suggested that delayed MNM-EGFP expression maintains chromosome conjunction in the chrXY bivalent more effectively than in autosomal bivalents, as indicated by the earlier analysis of fixed cells. As some chrXY missegregation was clearly revealed in *mnm(l)* by fixed cell analyses, the failure to detect abnormal chrXY behavior by time lapse imaging presumably reflects the far lower number of cells analyzed by this latter, more demanding method.

In conclusion, our phenotypic analyses of *mnm(l)* spermatocytes demonstrate that a delayed provision of MNM-EGFP, starting well after the formation of chromosome territories, restores homolog conjunction as well as faithful chromosome segregation during MI in *mnm* null mutants to a substantial extent but not completely. Moreover, conjunction and segregation of the sex chromosome bivalent is more normal in *mnm(l)* than that of the autosomes.

### Delayed SNM-EGFP expression does not rescue *snm* mutants

**Fig 5:**
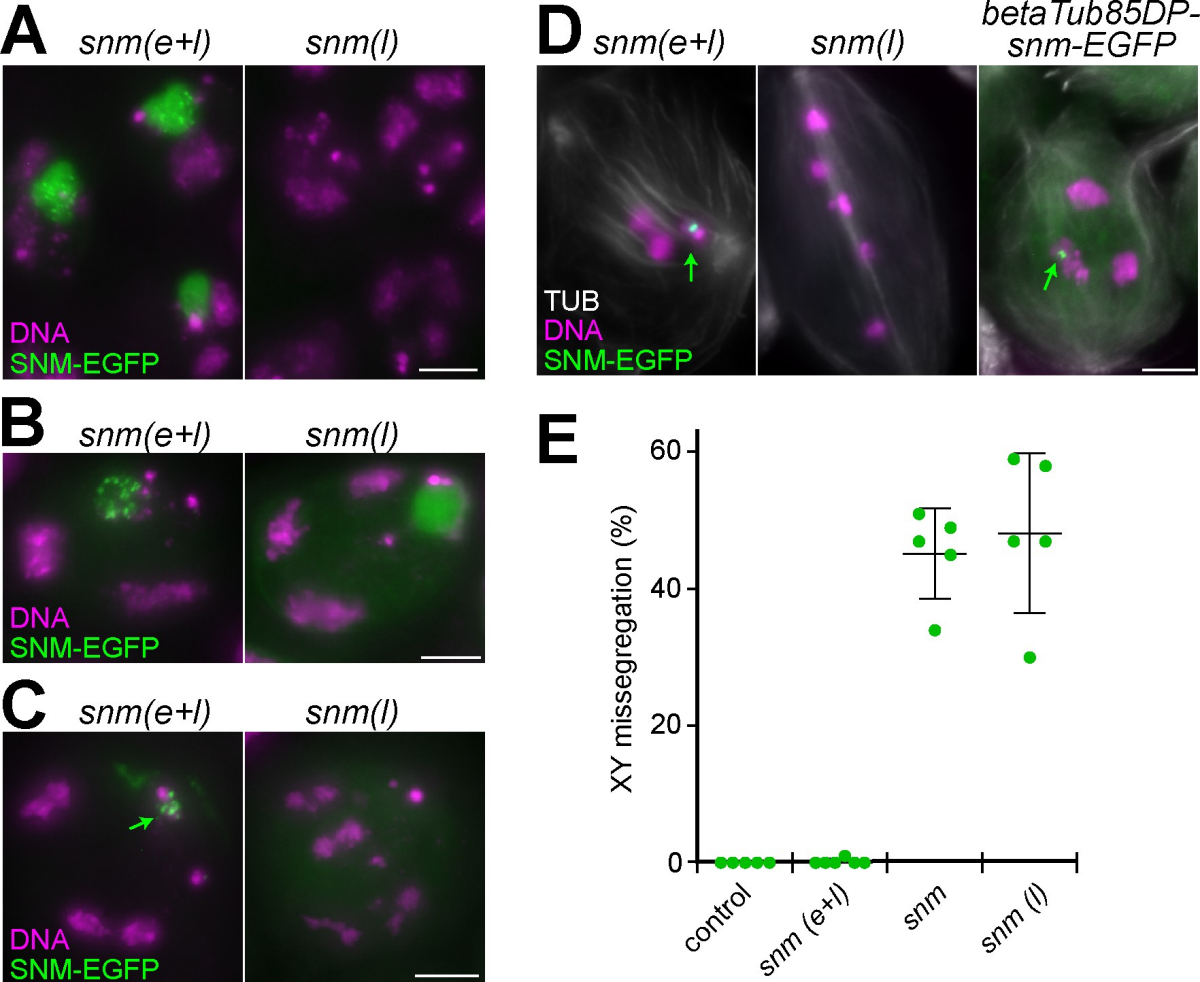
Delayed SNM-EGFP expression does not rescue *snm* mutants. To delay expression of SNM-EGFP until the late spermatocytes stages, a *betaTub85DP-snm-EGFP* transgene was introduced into *snm* null mutants. Expression pattern and phenotypic consequences were analyzed in testes isolated from the resulting *snm(l)* genotype. **(A)** At the S3 stage, SNM-EGFP is not yet detectable in *snm(l)* in contrast to *snm(e+l)*. **(B)** At the S5 stage, SNM-EGFP is present in both *snm(l)* and *snm(e+l)*, but subnucleolar foci are formed exclusively in the latter genotype. **(C)** At the S6 stage, SNM-EGFP foci (arrow) coalesce in *snm(e+l)*, while SNM-EGFP is dispersed throughout the nucleus in *snm(l)*. **(D)** During prometaphase I, a most prominent SNM-EGFP dot (arrow) on the chrXY bivalent, as in *snm(e+l)*, is absent in *snm(l)*, where also the number and size of chromosomes is abnormal. Localization of SNM-EGFP during prometaphase I is normal when *betaTub85DP-snm-EGFP* is in an *snm*^+^ background (*betaTub85DP-snm-EGFP*). **(E)** The rate of sex chromosome missegregation was assessed in early *snm(l)* spermatid cysts (n = 5) after XY FISH. For comparison, data from control, *snm(e+l)* and *snm* (see Fig 3D) is included. Average and s.d. are indicated. Bars = 5 μm.

To assess whether delayed expression restores chromosome conjunction and segregation during MI also in case of *snm*, a *betaTub85DP-snm-EGFP* transgene was made and introduced into the *snm* null mutant background. The resulting genotype will be designated as *snm(l)* in the following. Microscopic analyses confirmed that SNM-EGFP expression in *snm(l)* occurred with the expected delay in comparison to *snm(e+l)*. At the S3 stage (Fig 5A), where chromosome territories were already formed, SNM-EGFP was not yet detectable in *snm(l)*, while it was clearly present well before the S3 stage in *snm(e+l)*. At later stages, SNM-EGFP became detectable also in *snm(l)* spermatocytes (Fig 5B). However, its localization within the nucleolus was entirely diffuse, lacking the discrete subnucleolar foci of maximal signal intensity that were apparent in *snm(e+l)* (Fig 5B). In *snm(e+l)* spermatocytes, these SNM-EGFP foci coalesced into a single large dot on the chrXY bivalent during S6 (Fig 5C), when the nucleolus disintegrates and condensation of chromosome territories starts before the onset of the first meiotic divisions [17]. At the corresponding stage in *snm(l)*, SNM-EGFP was dispersed throughout the nucleus (Fig 5C). Similarly, during prometaphase I (Fig 5D), the characteristic strong SNM-EGFP dot on the chrXY bivalent was present in *snm(e+l)* cells but not in *snm(l)*. Prometaphase I spermatocytes with *betaTub85DP-snm-EGFP* in a *snm*^+^ background (rather than in an *snm* null background as in *snm(l)*) clearly displayed the prominent SNM-EGFP dot on the chrXY bivalent (Fig 5D), indicating that SNM-EGFP expressed by this particular transgene can localize normally in principle.

Apart from the absence of normal SNM-EGFP dots on the chrXY bivalent during prometaphase I in *snm(l)*, chromosome conjunction was observed to be completely defective in this genotype. In late spermatocytes, chromosome territories were either too many (Fig 5B and 5C) or they had a pronounced split appearance. During prometaphase I (Fig 5D), there were too many chromosome masses of smaller size compared to controls. These abnormalities in *snm(l)* were comparable to those displayed in *snm* null mutants (Fig 3C) [6]. In addition, quantitative analyses by XY FISH indicated that sex chromosome segregation during MI was random in *snm(l)* comparable to *snm* null mutants (Fig 5E). In conclusion, a delayed provision of SNM-EGFP is entirely insufficient for normal chromosome conjunction and segregation during MI, in contrast to our findings with MNM-EGFP (Fig 4) where substantial rescue had resulted after delayed expression.

### Delayed co-expression of SNM-EGFP and MNM-EGFP rescues *snm* mutants partially

Consideration of several additional findings and their interpretation suggested a potential explanation for the discrepant extent of rescue observed in *mnm(l)* and *snm(l)*. The original characterization of *mnm* and *snm* had already revealed interdependencies [6]. In *mnm* null spermatocytes, SNM was detected within the nucleolus as in wild-type, although it failed to form the strong dot on the sex chromosome bivalent eventually after disassembly of the nucleolus during entry into the first meiotic division [6]. In contrast, MNM could not be detected at any stage in *snm* null mutants [6]. Consistent with this latter finding, anti-MNM immunolabeling, which generates a characteristic dot-like signal readily detected on the chrXY bivalent during normal prometaphase I, failed to produce these signals, not only in *snm* null [6], but also in *snm(e)* and *snm(l)* mutants (S1 Fig). In *snm(e)*, the disappearance of anti-MNM signals occurred in parallel with SNM-EGFP degradation during the late spermatocyte stages, consistent with the notion that MNM protein is stabilized by SNM.

Additional analyses after transgenic *mnm-EGFP* expression confirmed this notion that *snm*^+^ gene function is required for MNM accumulation because SNM protein stabilizes MNM protein (rather than boosting *mnm* transcript levels). After *bamP-GAL4-VP16* driven *UASt-mnm-EGFP* expression in *snm* null mutants, MNM-EGFP was detected only very transiently in early spermatocytes outside of the nucleolus (S1 Fig). In contrast, after analogous *bamP-GAL4-VP16* driven *UASt-mnm-EGFP* expression in heterozygous siblings with a functional *snm*^+^ copy, MNM-EGFP translocated into the nucleolus and persisted there throughout spermatocyte maturation instead of disappearing rapidly after initial expression (S1 Fig).

Our finding that MNM did not detectably accumulate in *snm(l)* in parallel with SNM-EGFP during the late stages (S1 Fig) suggested that endogenous *mnm* transcripts might not be present any longer in late spermatocytes. However, if MNM synthesis normally occurs only transiently in early spermatocytes, the MNM detected during the late spermatocyte/MI stages would have to be protein resulting from early production and surviving owing to stabilization by SNM. Our experiments with *bamP-GAL4-VP16* driven *UASt-mnm-EGFP* expression actually indicated that MNM-EGFP protein can indeed stably perdure after early synthesis. *bamP-GAL4-VP16* is known to induce only a transient pulse of *UASt* transgene transcription in early spermatocytes, similar to the endogenous *bam* transcription pattern although with some delay known to be inherent to the GAL4/UAS system [28, 29]. The transient pulse of synthesis driven by *bamP-GAL4-VP16* in early spermatocytes is reported by MNM-EGFP generated after *UASt-mnm-EGFP* expression in *snm* mutants, where MNM-EGFP is highly unstable (S1 Fig). However, this transiently synthesized MNM-EGFP protein clearly perdures until MI after *bamP-GAL4-VP16* driven *UASt-mnm-EGFP* expression in the *snm*^*+*^ back ground (S1 Fig). Moreover, the complete rescue observed after *bamP-GAL4-VP16* driven *UASt-mnm-EGFP* in *mnm* mutants (i.e., *mnm(e+l)*) indicates that a transient pulse of MNM-EGFP synthesis in early spermatocytes is entirely sufficient for normal MI, arguing against occurrence and need for continuous MNM synthesis and exchange throughout spermatocyte maturation.

**Fig 6:**
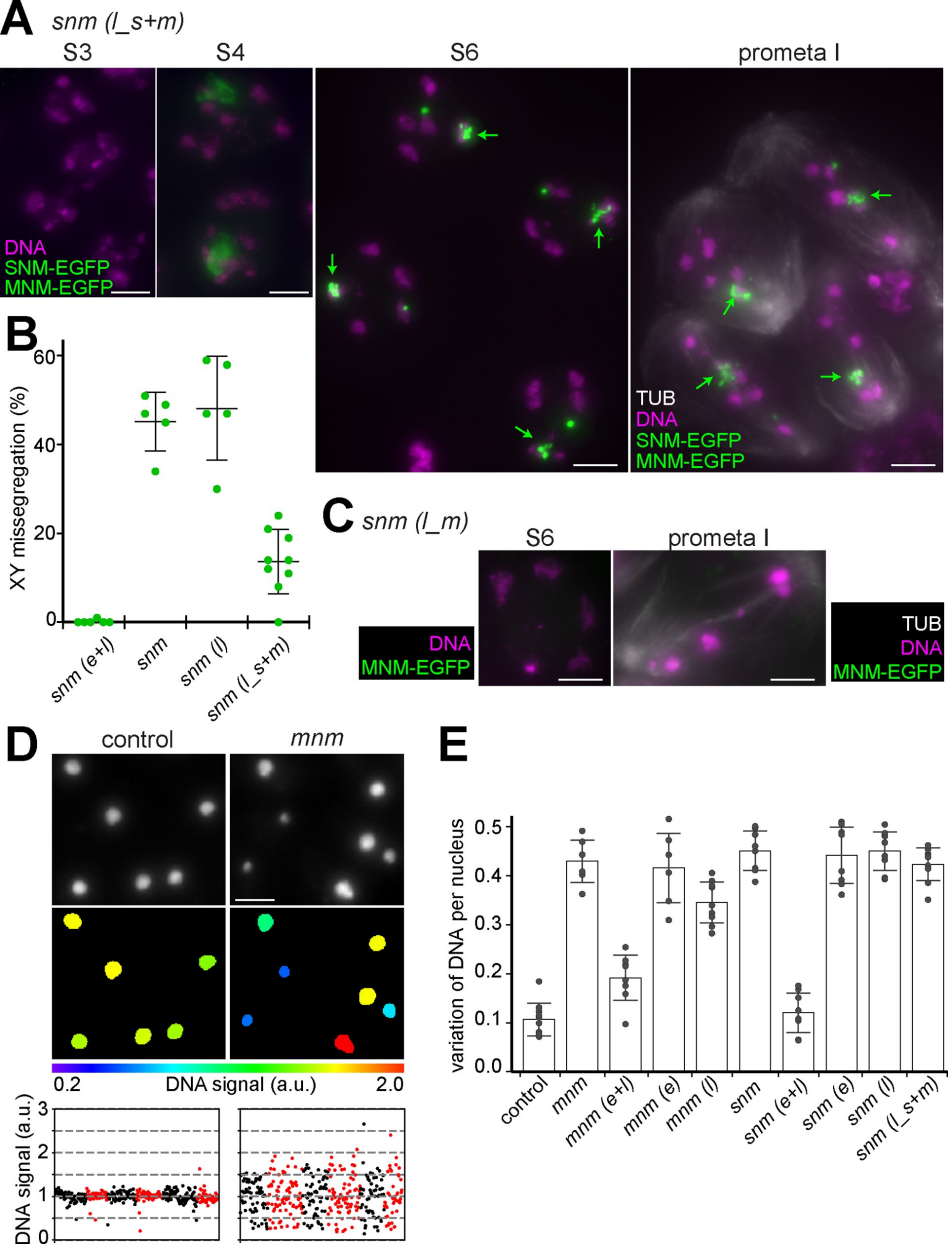
Partial rescue of *snm* mutants by delayed co-expression of both SNM-EGFP and MNM-EGFP. *betaTub85DP-snm-EGFP* and *betaTub85DP-mnm-EGFP* were introduced into *snm* null mutants. Expression pattern and phenotypic consequences were analyzed in testes isolated from the resulting *snm(l_s+m)* genotype. **(A)** EGFP signals and DNA staining at the indicated stages are displayed. At prometaphase I, anti-tubulin labeling used for the identification of this stage is shown as well. Arrows indicate cluster of EGFP foci associated with the chrXY bivalent. **(B)** The rate of sex chromosome missegregation was assessed in early *snm(l_s+m)* spermatid cysts (n = 9) after XY FISH. For comparison, data from *snm(e+l)*, *snm* and *snm(l)* (see Figs 3D and 5E) is included. Average and s.d. are indicated. **(C)** EGFP signals, DNA staining and anti-tubulin labeling at the indicated stages observed in *snm(l_m*) are displayed. **(D,E)** Chromosome missegregation during meiosis in the different genotypes was estimated by quantification of nuclear DNA signal intensities in early spermatid cysts. **(D)** Nuclei within maximum intensity projections of image stacks of early spermatid cysts (top) were identified semi-automatically and DNA signal intensities in these nuclei were quantified (middle). As illustrated by the data obtained from control and *mnm* null mutants, normal and random chromosome segregation during MI results in either low or high variation in the DNA content per spermatid nucleus, respectively (bottom). Values obtained for nuclei in a given cyst are plotted in the same color with different cysts from left to right alternating between black and red. **(E)** The coefficient of variation (CV) of the DNA signal intensity per nucleus was determined for at least six distinct spermatid cysts per genotype. Average of these CVs and s.d. are displayed for the indicated genotypes. Bars = 5 μm.

Based on the above considerations, it is hardly surprising that the delayed provision of SNM-EGFP in *snm(l)* does not rescue. When SNM-EGFP eventually accumulates in *snm(l)*, all endogenous MNM protein made in early spermatocytes is long degraded and endogenous *mnm* transcripts for a late re-accumulation of MNM appear to be absent. Without MNM, however, the SNM-EGFP which eventually accumulates in *snm(l)* cannot provide its function. This interpretation predicts that rescue of normal MI in *snm* null mutants by delayed provision of SNM-EGFP might succeed, if supported by concomitant delayed MNM-EGFP expression. To evaluate this possibility, we crossed both *betaTub85DP-mnm-EGFP* and *betaTub85DP-snm-EGFP* into *snm* null mutants. This genotype will be designated as *snm(l_s+m)*. As expected, EGFP signals were absent in in early *snm(l_s+m)* spermatocytes and EGFP accumulation started during the S4 stage primarily within the nucleolus (Fig 6A). Importantly, XY FISH analyses of early spermatid cysts clearly revealed that this delayed co-expression of both SNM-EGFP and MNM-EGFP in the *snm(l_s+m)* genotype reduced meiotic sex chromosome missegregation significantly in comparison to *snm* null and *snm(l)* (Fig 6B). Cytological analyses of spermatocytes provided further confirmation that the abnormalities in *snm(l_s+m)* were less severe than in *snm* null and in *snm(l)*. At the late S6 stage and during prometaphase I, each *snm(l_s+m)* spermatocyte displayed a single most prominent cluster of EGFP dots on one of the chromosome masses, i.e., the sex chromosome bivalent in all likelihood (Fig 6A). Additional weaker EGFP dots were present in these cells, and at least some of these were not associated with chromosome masses, presumably reflecting aggregates formed as a result of overexpression. Overall, our observations indicate that sex chromosome conjunction and segregation is largely rescued in *snm(l_s+m)*. In contrast, in *snm(l_m)*, a *snm* null genotype with delayed provision of only MNM-EGFP (but not SNM-EGFP), MNM-EGFP accumulation was actually not detectable and chromosome conjunction was completely defective during S6 and prometaphase I (Fig 6C). Therefore, rescue of sex chromosome conjunction in *snm* null mutants after delayed provision of conjunction proteins occurs only when SNM-EGFP and MNM-EGFP are co-expressed.

In contrast to sex chromosome conjunction, that of autosomes was still severely defective in the *snm(l_s+m)* spermatocytes. Already when EGFP accumulation started during the S4 stage, chromosome territories had an abnormal fragmented appearance (Fig 6A). Chromosome condensation during the S6 stage and during entry into MI exposed the defect in autosomal homolog conjunction very clearly (Fig 6A). The number of DNA masses in these late *snm(l_s+m)* spermatocytes was clearly increased compared to controls and their size was smaller (Fig 6A). This defect in autosomal homolog conjunction in *snm(l_s+m)* appeared to be just as severe as in *snm* null mutants (Fig 3B and 3C) or in *snm(l)* (Fig 5C and 5D).

Overall, our findings in *snm(l_s+m)* demonstrate that absence of SNM and MNM during the early spermatocyte stages followed by delayed provision of these proteins compromises autosomal conjunction more than sex chromosome conjunction. The *snm(l_s+m)* phenotype is therefore similar to the *mnm(l)* phenotype where autosomal conjunction was also observed to be more sensitive compared to sex chromosome conjunction.

For additional assessment of chromosome missegregation during meiosis in the different genotypes, we quantified nuclear DNA signals with image stacks of early spermatid cysts. In control testis, the 64 haploid nuclei within a given cyst are expected to have an almost identical DNA content given the negligible estimated size difference between chrX and chrY of around 0–10% of the total genome size. However, after random segregation of chromosomes during MI, as in *mnm* or *snm* null mutants, the DNA content of early spermatid nuclei is predicted to be far more variable. For an estimation of the DNA content in spermatid nuclei, we applied semi-automatic image analysis for quantification of the DNA staining intensity (Fig 6D). As predicted, the DNA signal intensities varied far less among early spermatid nuclei within control cysts compared to *mnm* and *snm* null mutant cysts (Fig 6D and S2 Fig). Therefore, as a measure of overall meiotic chromosome missegregation, we determined the coefficient of variation of nuclear DNA signal intensities for each cyst and calculated an average after analysis of at least 5 cysts per genotype. The comparison among the different genotypes (Fig 6E) confirmed that meiotic chromosome segregation was close to normal in *mnm(e+l)* and *snm(e+l)*. Severe defects comparable to those in *mnm* and *snm* null mutants were apparent in *mnm(e)*, *snm(e)* and *snm(l)*. In *mnm(l)*, chromosome segregation was less defective than in *mnm* null mutants (p < 0.004, t test) even though it was clearly not normal. In *snm(l_s+m)*, the overall chromosome missegregation appeared to be marginally less defective compared to *snm* null mutants (p < 0.17, t test), consistent with our results from cytological analysis of spermatocytes and XY FISH with spermatids in this genotype. The significant suppression of random segregation of sex chromosomes but not of autosomes results only in a subtle change in the variation of DNA content among spermatids in *snm(l_s+m)* compared to genotypes where missegregation affects all chromosomes.

## Discussion

The phenotypic consequences of a loss of MNM and SNM have clearly demonstrated that these proteins are required for normal conjunction of chromosomes into bivalents and hence for regular reductional segregation during MI in Drosophila spermatocytes [6]. These proteins might function directly as part of the physical linkage between chromosomes in bivalents. MNM and SNM are co-localized within a single prominent dot at the rDNA loci of the X and Y chromosomes [6], the known meiotic pairing sites of these otherwise highly heteromorphic sex chromosomes [13]. MNM and SNM disappear rapidly from the sex chromosome bivalent in a separase-dependent manner just before separation of the X and Y chromosomes to opposite spindle poles during the metaphase to anaphase transition of MI [6, 16, 24]. On autosomal bivalents, these proteins are more difficult to detect and the targeted chromosomal loci are unknown, but all phenotypic analyses have clearly argued for an analogous function in reductional autosome segregation during MI [6]. Here we provide further support that MNM and SNM are indeed part of the physical linkage between partner chromosomes within bivalents. These proteins are normally present throughout spermatocyte maturation during the stages S1-S6, as also the EGFP tagged versions expressed from *UASt* transgenes with the *bamP-GAL4-VP16* driver, which can replace the endogenous conjunction proteins functionally. Premature degradation after territory formation of either MNM-EGFP or SNM-EGFP in spermatocytes lacking the corresponding endogenous gene function results in an absence of conjunction and random chromosome segregation during MI. Conversely, delayed provision of these conjunction proteins after territory formation (under control of the *betaTub85D* regulatory region) is largely sufficient to restore conjunction and MI segregation of sex chromosomes, while rescue in case of autosomes is far less efficient.

Premature removal of the EGFP tagged conjunction proteins was achieved with the help of deGradFP [26]. This method is known to have variable efficiency with different GFP fusion proteins [26]. The method appears to degrade MNM-EGFP more efficiently than SNM-EGFP. The former but not the latter protein was lowered by our deGradFP transgene (*betaTub85DP-Nslmb-vhhGFP4*) already in early spermatocytes, presumably as a result of premature low level basal deGradFP expression. Moreover, MNM-EGFP could also no longer be detected during S6 and MI, while SNM-EGFP was still detectable during MI, although only at very low levels. While deGradFP reduced MNM-EGFP already during the early stages, compensating overexpression driven by *bamP-GAL4-VP16* from *UASt-mnm-EGFP* resulted in a level of remaining MNM-EGFP in *mnm(e)* that was comparable during the early stages to the level of endogenous MNM in wild type. Accordingly, the *mnm* null phenotype observed in *mnm(e)* indicates a requirement for MNM protein persistence until onset of anaphase I rather than a consequence of insufficient levels during the early stages. We acknowledge that this conclusion rests on comparisons of expression levels based on our microscopic quantification of signal intensities, an approach not free of pitfalls. In case of *snm(e)*, however, early reduction of SNM-EGFP did not occur. The very low SNM-EGFP levels still detectable during prometaphase I in *snm(e)* did not result in any rescue of homolog conjunction and regular MI segregation, indicating that normal MI in males clearly requires persistence of the SNM conjunction protein at high levels beyond territory formation.

Delayed provision of MNM-EGFP and SNM-EGFP in the corresponding null mutants was used for further delineation of the critical time period during which MNM and SNM have to be present for normal chromosome conjunction and regular MI segregation. In the relevant genotypes, *snm(l)* and *mnm(l)*, accumulation of the EGFP tagged conjunction proteins occurred after chromosome territory formation during the post-S3 stages. The resulting phenotypes indicate the critical role of temporal control of chromosome conjunction during spermatocyte maturation.

Time lapse imaging with *mnm(l)* spermatocytes indicated that delayed provision of MNM-EGFP is sufficient for normal chromosome conjunction and regular MI segregation. However, complete rescue was not observed consistently. Extensive phenotypic variability was also observed in fixed *mnm(l)* spermatocytes. Variability was observed between different cysts and to a lower degree also between spermatocytes within a cyst. Rescue of meiotic abnormalities varied from complete to none in *mnm(l)*. Moreover, a chromosome-specific difference in rescue efficiency was observed. Overall, conjunction and segregation of sex chromosomes was rescued more efficiently than that of the large autosomes. While we do not understand the cause of this variability, we propose the following explanation.

The relative temporal dynamics of MNM-EGFP accumulation driven by the *betaTub85D* regulatory region and of the forces responsible for chromosome territory formation might vary between spermatocyte cysts and lesser also within cysts. The molecular mechanisms that drive chromosome territory formation and control its temporal dynamics are still poorly understood. Condensin II activity is clearly required [30], but whether and how its activity might be regulated during spermatocyte maturation has not yet been studied. Interestingly, the reported effects of condensin II activity on polytene chromosomes in nurse cells and salivary glands [20, 31] indicate that it is likely responsible not only for the disruption of the non-homologous associations of pericentomeric hetereochromatin (i.e., the chromocenter) but also for the extensive unpairing of homologous euchromatic arms and of sister chromatids that accompanies chromosome territory formation during the S2b/S3 stage. Condensin II activity is likely to persist beyond this stage, as suggested by the observed increase of unpairing at some pericentromeric satellite loci and of homologous centromeres from S3 to S5 [19, 32]. We propose that MNM and SNM need to provide physical chromosome linkage throughout these stages in order to prevent condensin II activity from separating homologs completely. Accordingly, the chromosome territory expansion phenotype that develops in *mnm* and *snm* during the late spermatocyte stages [6] might result from the postulated continuing unpairing activity of condensin II. Delivery of MNM and SNM after a time point, at which homologs have separated completely, cannot re-establish pairing. In case of the rDNA loci containing sex chromosome bivalent, we propose that the forces which drive nucleolar assembly [33] provide independent resistance against unpairing by condensin II activity beyond MNM and SNM mediated conjunction. The latter might therefore become crucial once nucleolar disassembly starts during the late S5 stage [17]in preparation for entry into the first meiotic division [17]. Accordingly, the point of no return, after which provision of MNM-EGFP and SNM-EGFP can no longer achieve rescue, would be later in case of the sex chromosome bivalent compared to autosomal bivalents. Of course, alternative explanations for the observed higher rescue efficiency of sex chromosome conjunction in *mnm(l)* are not excluded. Overall, our observations suggest that MNM/SNM mediated linkage cannot establish initial chromosome pairing de novo. It can only fortify and maintain previously established associations and protect these against unpairing forces. Beyond the forces that drive territory formation (presumably condensin II), those that achieve chromosome condensation later during entry into the first meiotic division (presumably condensin I) and the spindle forces during bivalent bi-orientation during prometaphase I are additional unpairing forces which need to be counteracted to prevent premature bivalent splitting.

The phenotype observed in *snm(l_s+m)* provides additional support for the above proposal. This phenotype was similar but not identical to that of *mnm(l)*. While sex chromosome conjunction and regular MI segregation was rescued even somewhat better in *snm(l_s+m)* compared to *mnm(l)*, the opposite was recorded in case of the large autosomal bivalents. According to our proposal above, MNM and SNM mediated linkage appears to develop later in *snm(l_s+m)* than in *mnm(l)*, after the large autosomal time point of no return. The postulated delay might be linked to the program of *mnm* transcription and to MNM protein instability in the absence of SNM, as indicated by our analyses. Absence of MNM in *snm* mutants has been reported before [6]. Our results with MNM-EGFP expressed from transgenes argue that *snm*^+^ function promotes MNM accumulation by stabilizing MNM protein. The *bamP-GAL4-VP16* driver that we have used drives transient transcription of *UASt* transgenes restricted to the initial spermatocyte stages. Therefore, after *UASt-mnm-EGFP* expression with this *GAL4* driver, MNM-EGFP is detectable only very transiently in early spermatocytes if driver and target genes are in a *snm* null mutant background. In contrast, MNM-EGFP is present throughout spermatocyte maturation, if the same transgenes are in a *snm*^+^ background. As *snm*^+^ effects on the transcription program of the transgenes appear highly improbable, SNM protein seems to stabilize MNM protein. Consistent with this proposal, MNM was observed to disappear in parallel with SNM-EGFP in *snm(e)*. However, late provision of SNM-EGFP in *snm(l)* was not paralleled by MNM accumulation, indicating an absence of endogenous *mnm* transcripts in late spermatocytes. Therefore, in *snm(l_s+m)*, both SNM-EGFP and MNM-EGFP have to accumulate to effective concentrations without support by endogenous MNM before conjoining activity develops. In contrast, as endogenous SNM protein is present in *mnm* mutants[6] throughout the spermatocyte stages [6], conjoining activity by cooperation with the pre-existing endogenous SNM might develop more rapidly after late MNM-EGFP accumulation in *mnm(l)*, at least in some of the cysts where rescue of autosomal conjunction is observed.

While *bamP-GAL4-VP16* mediated *UASt-mnm-EGFP* transcription in the *mnm(e+l)* genotype is transient, MNM-EGFP is present throughout spermatocyte maturation, relying on stabilization by endogenous SNM. As the rescue of chromosome conjunction and regular MI segregation is essentially complete in *mnm(e+l)*, it follows that normal MI does not require continuous production of MNM throughout spermatocyte maturation. In wild-type, such a continuous MNM production is actually unlikely to occur, as endogenous *mnm* transcripts do not appear to be present in late spermatocytes. Therefore, we propose that MNM and SNM protein are integrated early into stable physical linker complexes that keep chromosomes paired.

Overall, our findings provide further support for the proposal that MNM/SNM mediated chromosome linkage provides a function comparable to cross-overs (COs) during canonical meiosis [2, 6]. After an initial homolog pairing that might rely on identical mechanisms in somatic and pre-meiotic cells in Drosophila, COs maintain this pairing during the canonical female meiosis and MNM/SNM mediated conjunction during the achiasmate male meiosis. However, a major difference between COs and MNM/SNM mediated conjunction presumably concerns linkage specificity. COs are generated by homologous recombination and therefore they are established exclusively between homologous chromosomes. In contrast, it is rather difficult to imagine how MNM/SNM-dependent linkage might discriminate accurately between homologous and non-homologous chromosome associations and develop specifically between the former, in particular in case of the autosomes. In the sex chromosomes, the rDNA loci function as pairing centers [34]. DNA sequences, perhaps via specific proteins that are present exclusively within the rDNA chromatin, might recruit MNM and SNM and thereby establish an appropriate linkage. However, the regions promoting autosomal pairing in spermatocytes appear to be distributed throughout the euchromatic arms and MNM/SNM appear to associate at a lower level at more than one site within autosomal territories. Even if MNM/SNM were recruited to specific loci (for which there is no evidence so far) what might prevent them from linking MNM/SNM recruiting positions on non-homologous chromosomes? In principle, the spermatocyte-specific process of territory formation, which separates the different bivalents apart, in combination with a temporally controlled activation of MNM/SNM-dependent linkage after territory formation (but not after the time point of no return), provides a solution compatible with the use of a non-discriminate linker. The essential temporal control of MNM/SNM-dependent linkage in this scenario cannot be explained by the pattern of *mnm* and *snm* expression. As in *snm(e+l)* and *mnm(e+l)*, endogenous MNM and SNM accumulation starts before territory formation [6]. An indiscriminate and effective MNM/SNM mediated chromosome linkage already during these early stages would be expected to inhibit the dissociation of the extensive non-homologous associations within the chromocenter by the process of chromosome territory formation. While MNM-EGFP and SNM-EGFP are clearly detectable before territory formation in *mnm(e+l)* and *snm(e+l)* spermatocytes, respectively, their initial subcellular localization is distinct from that observed during the later stages. Subnucleolar foci are absent initially; they form at around stage S2b and persist until they coalesce into a single sex chromosome bivalent associated dot in parallel with disassembly of the nucleolus and chromosome condensation during late S5 and S6. The formation of subnucleolar foci might therefore indicate when MNM/SNM-dependent stable linkage is activated.

In conclusion, the standard organization of chromosomes during interphase in Drosophila comprises the clustering of most pericentromeric heterochromatin into a single chromocenter in combination with isolation of paired homologous euchromatic regions into separate domains with minimal inter-homologous mingling [22]. Subsequently, during mitosis both the clustering of pericentromeric heterochromatin, as well as homolog pairing within the euchromatic regions, get disrupted by the processes of chromosome condensation and chromatid individualization, which are largely driven by condensin I [35], and by the spindle forces that interact with chromosomes for their eventual bi-orientation within the metaphase plate. To support achiasmate male meiosis, a mechanism seems to have evolved which preserves the homologous associations within the euchromatic regions in spermatocytes until after bi-orientation in metaphase I, but not the non-homologous associations within the pericentromeric heterochromatin. While our work effectively constrains the possibilities of how MNM and SNM contribute to this mechanism, various aspects of their proposed regulation and function remain speculative. The predictions made by our proposals will hopefully promote a successful future clarification.

## Materials and methods

### Drosophila lines

The following previously characterized mutant alleles and transgene insertions were used: *mnm*^*Z3-3298*^, *mnm*^*Z3-5578*^, *snm*^*Z3-0317*^, *snm*^*Z3-2138*^, *P{ry+*, *hsp70-mnm-EGFP}* [6]; *P{w*^*+*^, *bamP-GAL4-VP16}III* [25], *P{w*^*+*^, *His2Av-mRFP}II*.*2* and *P{w*^*+*^, *gCid-EGFP-Cid}II*.*1* [36]. The marked Y chromosome (*B*^S^*Yy*^+^) that was used for the genetic analysis of sex chromosome missegregation was obtained from *+/ B*^S^*Yy*^+^*; bw; mnm*^Z3-5578^*/TM3*, *Sb*, a stock kindly provided by Bruce McKee (University of Tennessee, Knoxville, TN, USA). Lines with the following transgenes were generated with the plasmid constructs described further below: *UASt-mnm*, *UASt-EGFP-mnm*, *UASt-mnm-EGFP*, *UASt-snm*, *UASt-EGFP-snm*, *UASt-snm-EGFP*, *betaTub85DP-mnm-EGFP*, *betaTub85DP-snm-EGFP*, and *betaTub85DP-Nslmb-vhhGFP4*. All transgenes under control of the cis-regulatory regions from *betaTub85D* were integrated into the attP40 landing site on chromosome 2L (25C6). Lines with combinations of mutant alleles and transgenes were generated by standard crosses. For all experiments, flies were cultured at 25°C. Detailed genotypes of the flies analyzed are provided in the supplemental material (S2 Table).

### Plasmids

For the production of transgenic lines allowing GAL4-dependent expression of *mnm* and *snm* with or without EGFP extensions we generated derivatives of pUASt [37], pUASt-EGFP-mcs or pUASt-mcs-EGFP [38]. The sequences of primers used for enzymatic amplification of insert fragments are provided in the supplemental material (S3 Table).

The inserts for *pUASt-mnm*, *pUASt-EGFP-mnm* and *pUASt-mnm-EGFP* were amplified from genomic DNA isolated from a *ry*^*506*^*; P{ry+*, *hsp70-mnm-EGFP}/CyO* male fly. The primer pair AB91/AB95 was used for *pUASt-mnm* and *pUASt-EGFP-mnm*; AB91/AB106 for *pUASt-mnm-EGFP*. After digestion with NotI, the insert fragments were cloned into the corresponding restriction site of the target vectors.

The inserts for *pUASt-snm*, *pUASt-EGFP-snm* and *pUASt-snm-EGFP* were amplified from a plasmid containing a full length *snm* cDNA (pGBD-SNM; kindly provided by Bruce McKee, University of Tennessee, Knoxville, TN, USA). The primer pair AB93/AB96 was used for *pUASt-snm* and *pUASt-EGFP-snm*; AB93/AB107 for *pUASt-snm-EGFP*. After digestion with NotI, the insert fragments were cloned into the corresponding restriction site of the target vectors.

For the production of transgenic lines that express the GFP-specific recombinant F box protein Nslmb-vhh4-GFP4 [26] under control of the cis-regulatory sequences of the spermatocyte-specific *betaTub85D* gene, we generated pattB-Nslmb-vhh4-GFP4, a pattB derivative [39]. Both 5’ and 3’ regions flanking the *betaTub85D* coding region (978 bp and 477 bp, respectively) were amplified from *w*^1^ genomic DNA and inserted into pattB. The region coding for Nslmb-vhh4-GFP4 was amplified from *pUASt-Nslmb-vhh4-GFP4* [26] and inserted in between the *betaTub85D* 5’ and 3’ flanking regions. The same *betaTub85D* cis-regulatory region was also used for the cloning of the *pattB-betaTub85DP-mnm-EGFP* and *pattB-betaTub85DP-snm-EGFP* constructs.

### Sex chromosome nondisjunction tests

Males of the genotype *w*/ *B*^S^*Yy*^+^*; UASt-xy/ +; allele-1/ allele-2 ± bamP-GAL4-VP16* were crossed to *w* virgin females. For the analyses where *UASt-xy* was *UASt-mnm II*.*1*, *UASt-EGFP-mnm II*.*1*, *UASt-mnm-EGFP II*.*1*, or *UASt-mnm-EGFP II*.*2*, allele-1 and allele-2 were *mnm*^Z3-5578^ and *mnm*^Z3-3298^, respectively. For the analyses where *UASt-xy* was *UASt-snm II*.*1*, *UASt-EGFP-snm II*.*1*, or *UASt-snm-EGFP II*.*1*, allele-1 and allele-2 were *snm*^Z3-2138^ and *snm*^Z3-0317^, respectively. Normal segregation of the sex chromosomes during male meiosis results in regular sperm with an X or a Y chromosome, while missegregation generates irregular sperm with either both an X and a Y chromosome or neither. The *B*^S^ marker allowed identification of progeny fathered by regular or irregular sperm, respectively. Fertilization of *w* oocytes with regular sperm results in XY males with Bar-eyes and XX females with normal eyes. In contrast, fertilization of *w* oocytes with irregular sperm results in X0 males with normal eyes or XXY females with Bar-eyes.

### Testis preparations

For whole mount testis preparations, dissection was performed in testis buffer (183 mM KCl, 47 mM NaCl, 10 mM Tris-HCl, pH 6.8). Testes were fixed in depression slides for 10 minutes in phosphate buffered saline (PBS) containing 4% formaldehyde and 0.1% Triton X-100. For DNA staining, testes were incubated for 10 minutes in PBS, 0.1% Triton X-100 (PBTx) containing 1 μg/ml Hoechst 33258. After three washes with PBS, testes were transferred into a drop of mounting medium (70% glycerol, 1% n-propyl gallate, 0.05% p-phenylenediamine, 50 mM Tris-HCl pH 8.5) on a slide before adding a cover slip.

Testis squash preparations were made and stained essentially as described previously [40], according to protocol 3.3.2, except that we used the mounting medium described above. For immunolabeling, mouse monoclonal anti-α-tubulin DM1A (Sigma) was used at 1:10000 and the rabbit antiserum against ModC [11] at 1:4000. This latter antibody recognizes the N-terminal region that is present in all of the many different isoforms expressed by the *mod(mdg4)* locus. However, as MNM appears to be the only *mod(mdg4)* protein product expressed in spermatocytes [10], the designation anti-MNM is used here. Secondary antibodies were Alexa568-conjugated goat antibodies against mouse or rabbit IgG diluted 1:1000.

For immuno-FISH, testes were dissected and fixed with 4% formaldehyde in PBS, followed by permeabilization with PBS containing 0.3% Triton X‐100 and 0.3% sodium deoxycholate. Anti-α-tubulin staining was done as described above except that Cy5-conjugated goat anti-mouse IgG diluted 1:1000 was used as secondary antibody. Ethanol incubations and dehydration with a formamide series were also done as described (immuno-FISH protocol 3.2, steps 10–26) [41]. An oligonucleotide (5'-TTTTCCAAATTTCGGTCATCAAATAATCAT-3') with Atto-565 on 5’ and 3’ end (Integrated DNA Technologies, Leuven, Belgium) was used for detection of the X-specific 359 bp repeats at a concentration of 1 ng/μl in hybridization buffer. An oligonucleotide (5'-AATACAATACAATACAATACAATACAATAC-3') with Alexa-488 on 5’ and 3’ end (Integrated DNA Technologies, Leuven, Belgium) was used for detection of the Y-specific AATAC satellite repeats at a concentration of 2 ng/μl in hybridization buffer. The denaturation step was performed at 98°C for 6 min, and hybridization over night at 18°C. Slides were washed twice for ten minutes each time in 50% formamide, 2x SSCT at 18°C. Thereafter, additional washes for ten minutes each time were performed at room temperature, first once in 25% formamide, 2x SSCT and then three times in 2x SSCT. DNA was stained with 1 μg/ml Hoechst 33258 for 10 minutes and slides were washed twice in PBS for 5 minutes before mounting.

### Microscopy and image analysis

Preparations were analyzed with a Zeiss Cell Observer HS microscope. For the quantification of DNA signal intensities in nuclei within early spermatid cysts, a 40×/0.75 oil immersion objective was used for acquisition of image stacks with 280 nm separation between focal planes. For high resolution images of spermatocytes, stacks with 250 nm spacing between focal planes were acquired using a 100×/1.4 oil immersion objective. The images displayed in the figures represent maximum intensity projections. The data used for statistical analyses of a particular genotype was obtained from multiple slides and each slide was prepared with about ten dissected testes.

The quantification of the intensity of the dot signals associated with the sex chromosome bivalent during prometaphase I after staining with anti-Mod(C) or resulting from MNM-EGFP was done using ImageJ software and subtraction of local background as described previously [32]. To minimize variability affecting quantification of anti-Mod(C) signal intensities, testes dissected from control and *mnm(e+l)* were combined onto the same slide for fixation and staining. During microscopic analysis, genotypes were assigned based on presence or absence of MNM-EGFP signals.

The quantification of the intensity of the DNA signal in nuclei within early spermatid cysts was performed with the help of a CellProfiler pipeline [42]. Within a maximum intensity projection of the acquired image stacks, the region containing nuclei of an early spermatid cyst at the onion stage was outlined manually. These regions did not necessarily contain all 64 spermatid nuclei of a given cyst but usually at least 50%. Nuclei of cysts cells within these regions were marked manually and eliminated from the analyses. For the plots documenting the variation of DNA signal intensity among the nuclei in early spermatid cysts (Fig 6D and S2 Fig), the DNA signal intensities of all the nuclei within the image stack obtained of a particular cyst were averaged. The resulting cyst average was then used for normalization of the DNA signal intensity values of the nuclei from this cyst. Thereafter, the standard deviation of these values for the cyst was determined. For the comparison between the different genotypes, the standard deviations of all the analyzed cysts of a given genotype were plotted (Fig 6E). Source data (including raw integrated intensity per spermatid nucleus) is provided in S4 Table.

### Time-lapse imaging

Time lapse imaging of progression through meiosis was performed as recently described [24]. In brief, testes from pupal or young adult males were dissected in Schneider’s Drosophila Medium (Invitrogen, #21720) supplemented with 10% fetal bovine serum (Invitrogen) and 1% penicillin/streptomycin (Invitrogen, #15140). The dissected testes were transferred into 40 μl of medium in a 35 mm glass bottom dish (MatTek Corporation, #P35G-1.5-14-C) and opened with fine tungsten needles to release the cysts. To reduce sample movements, 15 μl of 1% w/v methylcellulose (Sigma, #M0387) was added. A wet filter paper was placed inside along the dish wall before sealing the lid with parafilm. Imaging was performed at 25°C in a room with temperature control using a spinning disc confocal microscope (VisiScope with a Yokogawa CSU-X1 unit combined with an Olympus IX83 inverted stand and a Photometrics evolve EM 512 EMCCD camera, equipped for red/green dual channel fluorescence observation; Visitron systems, Puchheim, Germany) using a 60×/1.4 oil immersion objective. Image stacks with 24–30 focal planes spaced by 500 nm were acquired with a time interval of 10 or 20 seconds. Precise numbers are specified in the legends of S1 Movie and S2 Movie, respectively. Imaris software (Bitplane) was used to track centromere signals and for production of avi files from maximum intensity projections, as well as for exporting still frames, which were assembled using Photoshop (Adobe).

## Supporting information

S1 Fig Presence of MNM protein depends on *snm* function. **(A)** Squash preparations of testes of the indicated genotypes were immunolabeled with an antibody that detects MNM in spermatocytes and double labeled with a DNA stain. Spermatocytes in prometaphase I are displayed. The arrow indicates the strong anti-MNM dot signal that is present on the chrXY bivalent but only in the presence of *snm* function.
**(B)** *UASt-mnm-EGFP* was expressed with *bamP-GAL4-VP16* in either *snm* null mutants (bottom) or in the *snm* heterozygous siblings (top). EGFP signals and DNA staining are displayed within the apical regions of the testis tube. The most apical region with the germline stem cells and the hub cells is towards the left and differentiation proceeds towards the right. *bamP-GAL4-VP16* driven transcription is known to occur transiently during the late transit amplifying cycles (gonial division cycles). While MNM-EGFP signals are restricted to this transcribing region in *snm* mutants (bottom), these signals perdure to the late spermatocyte stages in the presence of *snm* function (top). Bars = 5 μm (A) and 50 μm (B).
(TIF)

S2 Fig Variation of DNA signal intensity per nucleus in early spermatid cysts. Nuclei within projections of image stacks of early spermatid cysts of the indicated genotypes were identified semi-automatically and DNA signal intensities in these nuclei were quantified. All values obtained within a given cyst were averaged and the average was used for normalization of the values. The normalized values obtained from a given cyst are plotted in the same color with different cysts of the same genotype arranged from left to right alternating between black and red. The different cysts within a given genotypes are ordered according to their coefficient of variation starting with the cyst characterized by the lowest variability on the left.
(TIF)

S1 Table Rescue of sex chromosome non-disjunction by *mnm* and *snm* variants expressed from *UASt* transgenes. (PDF)

S2 Table Description of the analyzed genotypes. (PDF)

S3 Table Primer sequences. (PDF)

S4 Table Source data for S2 Fig and Fig 6D and 6E. (XLSX)

S1 Movie Normal meiosis I in some *mnm(l)* spermatocytes. Progression through MI was analyzed by time lapse imaging with *mnm(l)* spermatocytes expressing histone His2Av-mRFP (magenta) and the centromere marker Cenp-A/Cid-EGFP (green) in addition to MNM-EGFP (green). With the display settings used during movie file production, only the strong MNM-EGFP dot on the chrXY bivalent is clearly detectable (until anaphase) but not the far weaker Cid-EGFP centromere signals. Bivalents and their segregation are normal in the *mnm(l)* spermatocyte shown in this movie.
(MP4)

S2 Movie Abnormal meiosis I with premature splitting of autosomal bivalents in other *mnm(l)* spermatocytes. Progression through MI was analyzed by time lapse imaging with *mnm(l)* spermatocytes expressing histone His2Av-mRFP (grey) and the centromere marker Cenp-A/Cid-EGFP (green) in addition to MNM-EGFP (green). With the display settings used during movie file production, only the strong MNM-EGFP dot on the chrXY bivalent is clearly detectable (until anaphase) but not the far weaker Cid-EGFP centromere signals. However, centromere positions are indicated by small colored spheres in the *mnm(l)* spermatocyte shown in this movie. The pink and red spheres mark the centromeres of chrX and chrY, respectively. Yellow spheres mark autosomal centromeres. During metaphase at the start of the movie, only the chrXY bivalent is normally conjoined and aligned at the metaphase plate, while the autosomes are present already as univalents and off the metaphase plate.
(MP4)
