# Supplementary figures and images for: MNM and SNM maintain but do not establish achiasmate homolog conjunction during Drosophila male meiosis
Source: PLoS Genet. 2019 May 28;15(5):e1008162. doi: 10.1371/journal.pgen.1008162 (PMC6538143; doi:10.1371/journal.pgen.1008162)

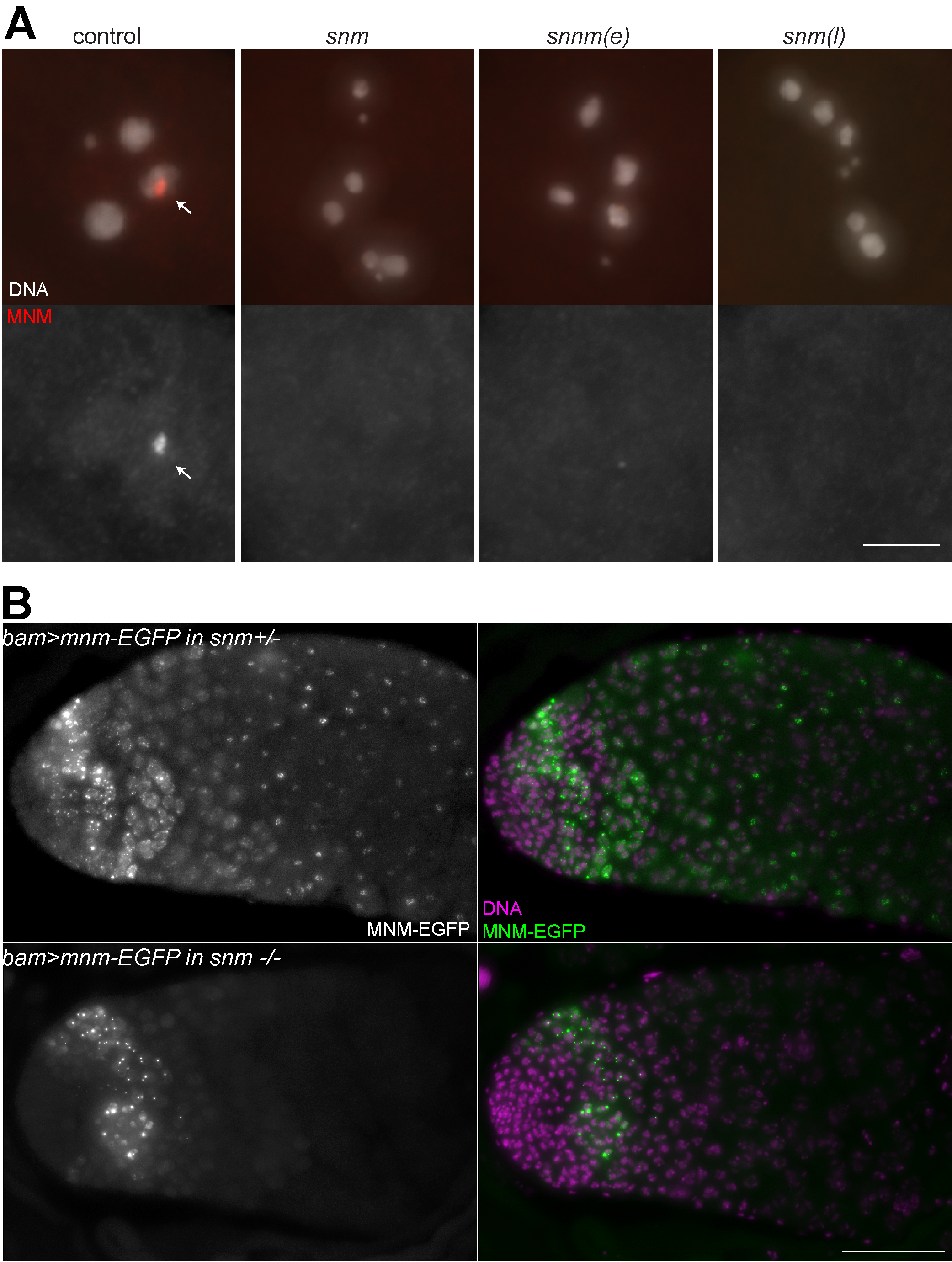

Supplement: S1 Fig — (A) Squash preparations of testes of the indicated genotypes were immunolabeled with an antibody that detects MNM in spermatocytes and double labeled with a DNA stain. Spermatocytes in prometaphase I are displayed. The arrow indicates the strong anti-MNM dot signal that is present on the chrXY bivalent but only in the presence of snm function. (B) UASt-mnm-EGFP was expressed with bamP-GAL4-VP16 in either snm null mutants (bottom) or in the snm heterozygous siblings (top). EGFP signals and DNA staining are displayed within the apical regions of the testis tube. The most apical region with the germline stem cells and the hub cells is towards the left and differentiation proceeds towards the right. bamP-GAL4-VP16 driven transcription is known to occur transiently during the late transit amplifying cycles (gonial division cycles). While MNM-EGFP signals are restricted to this transcribing region in snm mutants (bottom), these signals perdure to the late spermatocyte stages in the presence of snm function (top). Bars = 5 μm (A) and 50 μm (B). (TIF) [file pgen.1008162.s001.tif]

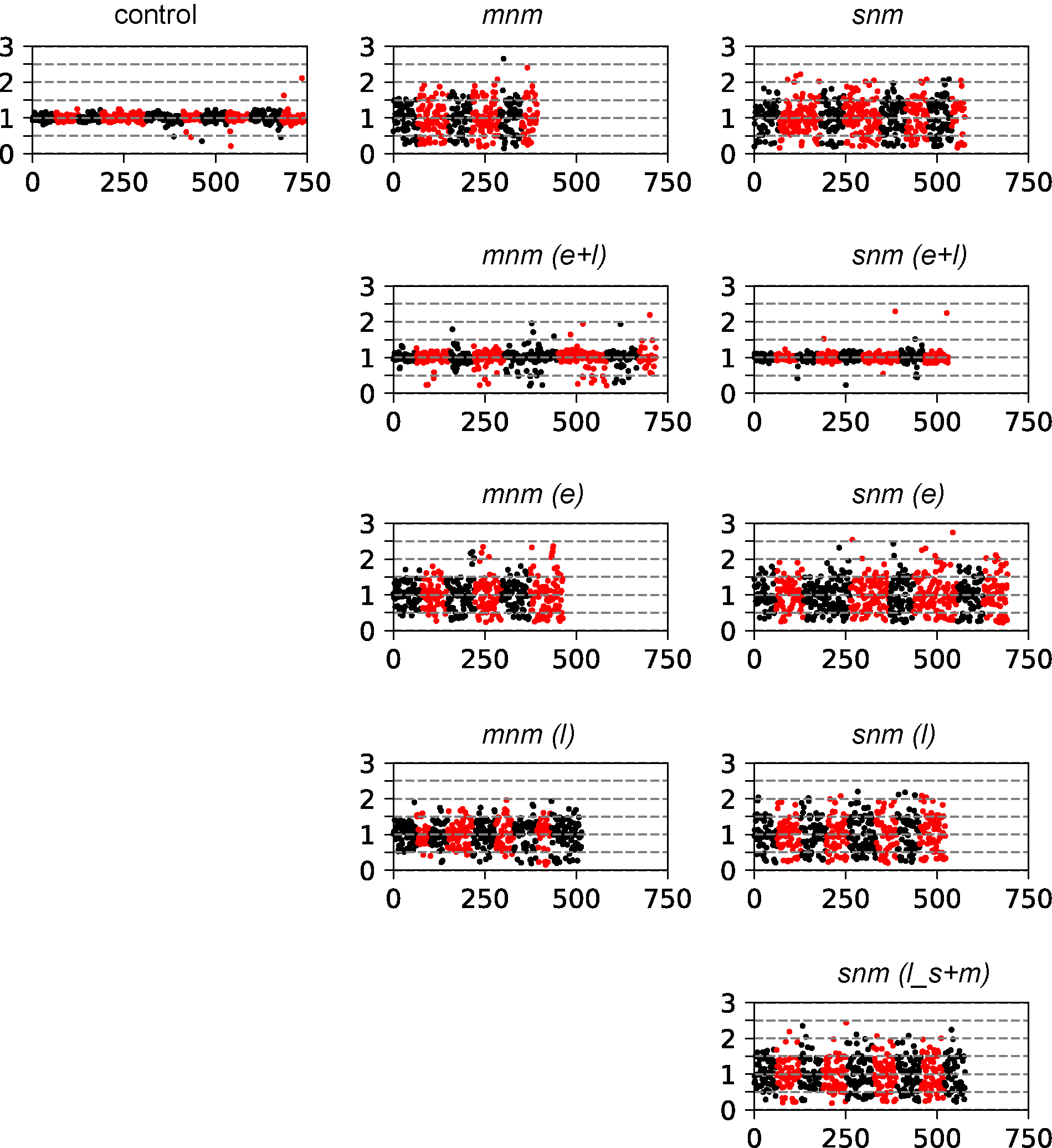

Supplement: S2 Fig — Nuclei within projections of image stacks of early spermatid cysts of the indicated genotypes were identified semi-automatically and DNA signal intensities in these nuclei were quantified. All values obtained within a given cyst were averaged and the average was used for normalization of the values. The normalized values obtained from a given cyst are plotted in the same color with different cysts of the same genotype arranged from left to right alternating between black and red. The different cysts within a given genotypes are ordered according to their coefficient of variation starting with the cyst characterized by the lowest variability on the left. (TIF) [file pgen.1008162.s002.tif]
