# Supplementary material for: MNM and SNM maintain but do not establish achiasmate homolog conjunction during Drosophila male meiosis
Source: PLoS Genet. 2019 May 28;15(5):e1008162. doi: 10.1371/journal.pgen.1008162 (PMC6538143; doi:10.1371/journal.pgen.1008162)
Supplement: S1 Table — (PDF) [file pgen.1008162.s003.pdf]

S1 Table. Rescue of sex chromosome non-disjunction by *mnm* and *snm* variants expressed from *UASt* transgenes

genotype [a)]	number of regular adult progeny [b)]		number of irregular adult progeny [c)]		sex chromosome non-disjunction (%) [d)]
	m	f	m	f	m + f
control	399	508	1	2	0.3
mnm	120	153	204	51	48.3
mnm + UASt-mnm II.1	182	207	214	96	44.3
mnm + UASt-mnm II.1 + bam	605	739	5	1	0.4
mnm + UASt-EGFP-mnm II.1	195	215	275	93	47.3
mnm + UASt-EGFP-mnm II.1 + bam	498	612	73	42	9.4
mnm + UASt-mnm-EGFP II.1 [e)]	951	1278	18	4	1.0
mnm + UASt-mnm-EGFP II.1 + bam	949	1205	82	50	0.6
mnm + UASt-mnm-EGFP II.2	154	180	233	65	47.2
mnm + UASt-mnm-EGFP II.2 + bam	407	410	5	3	1.0
snm	133	153	191	84	49.0
snm + UASt-snm II.1	162	190	225	110	48.8
snm + UASt-snm II.1 + bam	650	708	14	2	1.2
snm + UASt-EGFP-snm II.1	160	168	197	106	48.0
snm + UASt-EGFP-snm II.1 + bam	373	544	13	3	1.7
snm + UASt-snm-EGFP II.1	131	155	193	70	47.9
snm + UASt-snm-EGFP II.1 + bam	558	645	2	0	0.2

a) All analyzed males had $B^S Yy^+$, a marked Y-chromosome used for the analysis of the frequency of sex chromosome non-disjunction. Fertilization of euploid oocytes with irregular nullo-sperm (without X or Y) or diplo-sperm (with both X and Y) results in X0 males with normal eyes and XXY females with Bar eyes, respectively. In contrast, the combination of regular sperm (with either X or Y) and euploid oocytes results in phenotypically distinct XY males with Bar eyes and XX females with normal eyes. “control” indicates the presence of two wild-type copies of both *mnm* and *snm*. ”*mnm*” indicates $mnm^{Z3\text{-}5578}/mnm^{Z3\text{-}3298}$. “*snm*” indicates $snm^{Z3\text{-}0317}/snm^{Z3\text{-}2138}$. “*bam*” indicates *bamP-GAL4-VP16 (III).*
b) Males with the listed genotypes were crossed to *w* virgin females followed by analysis of adult F1 progeny. The number of male (m) and female (f) F1 progeny resulting from regular sperm is indicated.
c) The number of male (m) and female (f) F1 progeny resulting from irregular sperm is indicated.
d) Overall sex chromosome non-disjunction (%) corresponding to the number of irregular progeny (m + f) divided by the total number of progeny (m + f) is indicated.
e) The *bamP-GAL4-VP16* independent suppression of sex chromosome missegregation observed with *UASt-mnm-EGFP II.1* might reflect high basal expression of this transgene insertion caused by an insertion position effect.
