# Supplementary material for: MNM and SNM maintain but do not establish achiasmate homolog conjunction during Drosophila male meiosis
Source: PLoS Genet. 2019 May 28;15(5):e1008162. doi: 10.1371/journal.pgen.1008162 (PMC6538143; doi:10.1371/journal.pgen.1008162)
Supplement: S2 Table — (PDF) [file pgen.1008162.s004.pdf]

S2 Table. Description of the analyzed genotypes

Fig 1A	F1 progeny was analyzed obtained from crosses of w^1 virgin females with males of the following genotypes:<br>control: w^1/ B^SYy^+<br>*mnm*: *w**/ B^SYy^+; +/ +; $mnm^{Z3\text{-}3298}$/ $mnm^{Z3\text{-}5578}$<br>*mnm (e+l)*: *w**/ B^SYy^+; *UASt-mnm-EGFP* II.2/ +; *bamP-GAL4-VP16*, $mnm^{Z3\text{-}3298}$/ $mnm^{Z3\text{-}5578}$<br>*snm*: *w**/ B^SYy^+; +/ +; $snm^{Z3\text{-}0317}$/ $snm^{Z3\text{-}2138}$<br>*snm (e+l)*: *w**/ B^SYy^+; *UASt-snm-EGFP* II.1/ +; *bamP-GAL4-VP16*, $snm^{Z3\text{-}0317}$/ $snm^{Z3\text{-}2138}$
Fig 1B	control: *w**/ *Y*; +/ +; *bamP-GAL4-VP16*/ +
Fig 1C,D	control: w^1/ *Y*<br>*mnm (e+l)*: *w**/ *Y*; *UASt-mnm-EGFP* II.2/ +; *bamP-GAL4-VP16*, $mnm^{Z3\text{-}3298}$/ $mnm^{Z3\text{-}5578}$<br>*snm (e+l)*: *w**/ *Y*; *UASt-snm-EGFP* II.2/ +; *bamP-GAL4-VP16*, $snm^{Z3\text{-}0317}$/ $snm^{Z3\text{-}2138}$
Fig 2	control: w^1/ *Y*<br>*mnm*: *w**/ *Y*; +/ +; *bamP-GAL4-VP16*, $mnm^{Z3\text{-}3298}$/ $mnm^{Z3\text{-}5578}$<br>*mnm (e)*: *w**/ *Y*; *UASt-mnm-EGFP* II.1, *betaTub85DP-Nslmb-vhhGFP4*/ +; *bamP-GAL4-VP16*, $mnm^{Z3\text{-}3298}$/ $mnm^{Z3\text{-}5578}$<br>*mnm (e+l)*: *w**/ *Y*; *UASt-mnm-EGFP* II.2/ +; *bamP-GAL4-VP16*, $mnm^{Z3\text{-}3298}$/ $mnm^{Z3\text{-}5578}$
Fig 3	control: w^1/ *Y*<br>*snm*: *w**/ *Y*; +/ +; *bamP-GAL4-VP16*, $snm^{Z3\text{-}0317}$/ $snm^{Z3\text{-}2138}$<br>*snm (e)*: *w**/ *Y*; *UASt-snm-EGFP* II.2, *betaTub85DP-Nslmb-vhhGFP4*/ +; *bamP-GAL4-VP16*, $snm^{Z3\text{-}0317}$/ $snm^{Z3\text{-}2138}$<br>*snm (e+l)*: *w**/ *Y*; *UASt-snm-EGFP* II.2/ +; *bamP-GAL4-VP16*, $snm^{Z3\text{-}0317}$/ $snm^{Z3\text{-}2138}$

Fig 4A-D	control: w^{1}/ Y<br><br>mnm: w*/ Y; +/ +; bamP-GAL4-VP16, mnm$^{Z3-3298}$/ mnm$^{Z3-5578}$<br><br>mnm (e+l): w*/ Y; UASt-mnm-EGFP II.2/ +; bamP-GAL4-VP16, mnm$^{Z3-3298}$/ mnm$^{Z3-5578}$<br><br>mnm (l): w*/ Y; betaTub85DP-mnm-EGFP/ +; mnm$^{Z3-3298}$/ mnm$^{Z3-5578}$
Fig 4E-G	mnm (l): w*/ Y; betaTub85DP-mnm-EGFP/ His2Av-mRFP II.2, gCid-EGFP-Cid II.1; mnm$^{Z3-3298}$/ mnm$^{Z3-5578}$
Fig 5	control: w^{1}/ Y<br><br>snm: w*/ Y; +/ +; bamP-GAL4-VP16, snm$^{Z3-0317}$/ snm$^{Z3-2138}$<br><br>snm (e+l): w*/ Y; UASt-snm-EGFP II.2/ +; bamP-GAL4-VP16, snm$^{Z3-0317}$/ snm$^{Z3-2138}$<br><br>snm (l): w*/ Y; betaTub85DP-snm-EGFP/ +; snm$^{Z3-0317}$/ snm$^{Z3-2138}$<br><br>betaTub85DP-snm-EGFP: w*/ Y; betaTub85DP-snm-EGFP/ CyO; snm$^{Z3-0317}$/ TM6B
Fig 6A-C	snm: w*/ Y; +/ +; bamP-GAL4-VP16, snm$^{Z3-0317}$/ snm$^{Z3-2138}$<br><br>snm (l): w*/ Y; betaTub85DP-snm-EGFP/ +; snm$^{Z3-0317}$/ snm$^{Z3-2138}$<br><br>snm (l_m): w*/ Y; betaTub85DP-mnm-EGFP/ +; snm$^{Z3-0317}$/ snm$^{Z3-2138}$<br><br>snm (l_s+m): w*/ Y; betaTub85DP-snm-EGFP/ betaTub85DP-mnm-EGFP; snm$^{Z3-0317}$/ snm$^{Z3-2138}$

Fig 6D,E	control: *w*^{1}/ *Y*<br>*mnm*: *w*/ Y*; +/ +; *bamP-GAL4-VP16, mnm*$^{Z3-3298}$/ *mnm*$^{Z3-5578}$<br>*mnm (e+l)*: w*/ *Y*; *UASt-mnm-EGFP* II.2/ +; *bamP-GAL4-VP16, mnm*$^{Z3-3298}$/ *mnm*$^{Z3-5578}$<br>*mnm (e)*: w*/ *Y*; *UASt-mnm-EGFP* II.1, *betaTub85DP-Nslmb-vhhGFP4*/ +; *bamP-GAL4-VP16, mnm*$^{Z3-3298}$/ *mnm*$^{Z3-5578}$<br>*mnm (l)*: *w*/ Y*; *betaTub85DP-mnm-EGFP*/ +; *mnm*$^{Z3-3298}$/ *mnm*$^{Z3-5578}$<br>*snm*: *w*/ Y*; +/ +; *bamP-GAL4-VP16, snm*$^{Z3-0317}$/ *snm*$^{Z3-2138}$<br>*snm (e+l)*: *w*/ Y*; *UASt-snm-EGFP* II.2/ +; *bamP-GAL4-VP16, snm*$^{Z3-0317}$/ *snm*$^{Z3-2138}$<br>*snm (e)*: *w*/ Y*; *UASt-snm-EGFP* II.2, *betaTub85DP-Nslmb-vhhGFP4*/ +; *bamP-GAL4-VP16, snm*$^{Z3-0317}$/ *snm*$^{Z3-2138}$<br>*snm (l)*: *w*/ Y*; *betaTub85DP-snm-EGFP*/ +; *snm*$^{Z3-0317}$/ *snm*$^{Z3-2138}$<br>*snm (l_s+m)*: *w*/ Y*; *betaTub85DP-snm-EGFP*/ *betaTub85DP-mnm-EGFP*; *snm*$^{Z3-0317}$/ *snm*$^{Z3-2138}$
S1 Fig	control: *w*^{1}/ *Y*<br>*snm*: *w*/ Y*; +/ +; *bamP-GAL4-VP16, snm*$^{Z3-0317}$/ *snm*$^{Z3-2138}$<br>*snm (e)*: *w*/ Y*; *UASt-snm-EGFP* II.2, *betaTub85DP-Nslmb-vhhGFP4*/ +; *bamP-GAL4-VP16, snm*$^{Z3-0317}$/ *snm*$^{Z3-2138}$<br>*snm (l)*: *w*/ Y*; *betaTub85DP-snm-EGFP*/ +; *snm*$^{Z3-0317}$/ *snm*$^{Z3-2138}$<br>*bam>mnm-EGFP in snm+/-*: w*/ *Y*; *UASt-mnm-EGFP* II.2/+; *bamP-GAL4-VP16, snm*$^{Z3-0317}$/ TM3, Ser<br>*bam>mnm-EGFP in snm-/-*: w*/ *Y*; *UASt-mnm-EGFP* II.2/+; *bamP-GAL4-VP16, snm*$^{Z3-0317}$/ *snm*$^{Z3-2138}$
S2 Fig	see Fig 6D,E
S1 Movie<br>S2 Movie	see Fig 4E-G
