# Supplementary material for: MNM and SNM maintain but do not establish achiasmate homolog conjunction during Drosophila male meiosis
Source: PLoS Genet. 2019 May 28;15(5):e1008162. doi: 10.1371/journal.pgen.1008162 (PMC6538143; doi:10.1371/journal.pgen.1008162)
Supplement: S3 Table — (PDF) [file pgen.1008162.s005.pdf]

S3 Table. Primer sequences

designation	sequence (5'- ... - 3')
AB91	CAAGGCGGCCGCAATGGCGGACGACGAGCAATTC
AB93	CAAGGCGGCCGCAATGAGTGATATATCTTTTGAT
AB95	CATTGCGGCCGCCTACAAATGGTTGTGCACGCC
AB96	CATTGCGGCCGCTCAAGTTGCCTGAGGATAAGA
AB106	CATTGCGGCCGCGCCAAATGGTTGTGCACGCCTGA
AB107	CATTGCGGCCGCGCAGTTGCCTGAGGATAAGATTT
